# Multifaceted insights of experimental, surface, and computational investigations for a synthesized pyrazolyl derivative inhibitor for carbon steel corrosion in an acidic environment

**DOI:** 10.1038/s41598-025-28496-3

**Published:** 2025-12-04

**Authors:** Basma M. A. Khedr, Sayed K. Ramadan, Sherine A. Abdelkader, Samar Abdelhamed, Mona A. El-Etre, A. Elaraby, Magdy A. M. Ibrahim

**Affiliations:** 1 Chemistry Department, Faculty of Science, Ain Shams University, Cairo, 11566 Egypt; 2 Basic Engineering Sciences Department, Faculty of Engineering, Benha University, Benha, Egypt; 3 Department of Basic Science, Faculty of Engineering at Shoubra, Benha University, Benha, Egypt; 4 Egyptian Petroleum Research Institute, Cairo,Nasr City, 11727 Egypt

**Keywords:** Corrosion inhibitor, Pyrazole, EIS, Langmuir isotherm, DFT, Adsorption, Chemistry, Materials science

## Abstract

For sustainable corrosion protection, this study introduces newly synthesized pyrazolyl-*N*-acetylthiocarbohydrazone (*PTH*) as a highly efficient and environmentally friendly inhibitor for carbon steel (CS) in an aggressive 1.0 M HCl solution. A comprehensive evaluation of *PTH* inhibition performance was conducted through chemical weight loss and electrochemical techniques, involving potentiodynamic polarization (*PDP*) and electrochemical impedance spectroscopy (*EIS*), which demonstrated a significant reduction in *CS* corrosion. *PDP* and *EIS* reinforced these findings, confirming the robust protective nature of *PTH* with an inhibition potency of 96%. The mitigation power of the *PTH* can be explained by its adsorption onto the *CS* surface, which followed the Langmuir adsorption model. The inhibitor exhibited exceptional stability and efficiency across varying temperature conditions and various immersion times using EIS, reinforcing its reliability in harsh acidic media, with a mitigation capacity of 97.16% at 50 °C and 97.3% after 24 h. The morphology of the *CS* surface was examined using *SEM* /EDX (Scanning Electron Microscopy), *AFM* (Atomic Force Microscopy), and *XPS* (X-ray Photoelectron Spectroscopy), exhibiting the *PTH* adsorption over *CS*, which was also proved and elucidated employing theoretical quantum investigations as density functional theory and Monte Carlo simulations.

## Introduction

 Corrosion is an inevitable and persistent challenge for metallic materials, manifesting as a complex electrochemical degradation process that progressively compromises structural integrity and functionality^1–3^. This phenomenon poses a critical threat across various industrial domains, including infrastructure, chemical manufacturing, wastewater treatment, and the petroleum sector, where metal components are subjected to aggressive corrosive environments^4,5^. Among the most applied materials, carbon steel is highly susceptible to corrosion, primarily due to chloride-induced de-passivation and carbonation effects in industrial and marine settings^6–8^. The use of acids in the industry, in descaling and oil well acidizing, leads to the gradual dissolution of the metal. The dissolution of metal can be minimized by surface metal modification or by making the surrounding environment of the exposed metal less severe or destructive. In economic terms, the huge damage resulting from the corrosion process in the petroleum field is more than 3% of the Press Information Bureau (PIB) in the USA. The annual direct cost of metallic corrosion ranges from 2% to 4% of the gross domestic product (GDP) in industrialized countries, and the trend is becoming higher and higher in the future^9,10^.

Additionally, carbon steel deterioration is a major concern in oil refineries, chemical plants, and ore extraction facilities, where acid pickling and exposure to highly aggressive media accelerate the breakdown of the metal matrix^11^. While absolute corrosion prevention remains unattainable, its progression can be significantly suppressed through advanced protective strategies, including alloy engineering, surface functionalization, cathodic protection, and the deployment of high-performance corrosion inhibitors^12–14^. Among these, corrosion inhibitors stand out as a particularly effective and operate through interfacial adsorption and protective film formation, thereby mitigating metal dissolution and surface degradation^15,16^. In recent years, pyrazole-based derivatives have garnered substantial attention in both industrial and medicinal applications due to their versatile electronic properties, structural adaptability, and pronounced coordination capabilities^17–19^. Notably, these compounds exhibit remarkable anti-corrosive potential, attributed to their ability to establish strong coordination interactions between their nitrogen-rich moieties and the vacant d-orbitals of metal atoms, leading to the formation of a robust protective barrier on metallic surfaces^20^. The efficiency of these inhibitors is inherently governed by their electronic density distribution, molecular conformation, functional group polarity, and steric effects, which dictate their adsorption characteristics and overall inhibition performance^21–23^.

The study by Abdelmalek et al.^24^. presented the synthesis and testing of a pyrazole derivative (*BM-01*) as a *CS* corrosion mitigator immersed in acidic 1.0 M HCl using *WL* (weight loss), *PDP* (potentiodynamic polarization), and *EIS* (electrochemical impedance spectroscopy), reflecting that the inhibition power was about 90%. In addition, *BM-01* acted as a mixed-type mitigator with dominant cathodic behavior. *SEM* (Scanning Electron Microscopy) and theoretical (*DFT*, *MD*) analyses confirmed the strong chemical adsorption and protective film formation of *BM-01*. Khalid et al.^25^, reported the synthesis and evaluation of a corrosion inhibitor based on a pyrazole derivative for *CS* in 1.0 M HCl, *via* electrochemical analyses, revealed that the examined inhibitor achieves up to 97.2% inhibition efficiency at optimal concentration, which can be clarified through the adsorption behavior that followed the Langmuir isotherm, indicating a strong chemisorption process.

Also, complementary theoretical evaluation, including *DFT* and molecular dynamics simulations, confirmed the inhibitor’s strong binding affinity and stable adsorption mechanism. Yurt et al.^26^. investigated the effectiveness of several Schiff base compounds as corrosion inhibitors for carbon steel in 0.1 M HCl using *PDP* and AC impedance spectroscopy, with significant inhibition reaching up to 92%. The studied inhibitors acted primarily as anodic-type, forming adsorbed protective films on the metal surface through their adsorption that followed the Temkin isotherm with a chemisorption process. In this context, a novel corrosion mitigator (*PTH*) abundant with various active sites in its molecular structure as hetero atoms (N, O, and S), π-electrons in C = N, C = O, and C = S, besides aromatic rings, which facilitated *PTH* adsorption onto the *CS* surface, forming a stable protective layer against the destructive agents. Comprehensive multi-techniques such as *WL*, *PDP*, and *EIS*, besides, *CS* surface examination was also applied utilizing *SEM*, *EDX* (Energy Dispersive X-ray Spectroscopy), *AFM* (Atomic Force Microscopy), and *XPS* (X-ray Photoelectron Spectroscopy) for more information about *PTH* mitigation performance. Finally, computational modeling as *DFT* (density functional theory) and *MCs* (Monte Carlo simulations), was employed to discuss the mechanistic aspects of *PTH* adsorption.

## Materials and methods

### Inhibitor synthesis

**Fig. 1:**
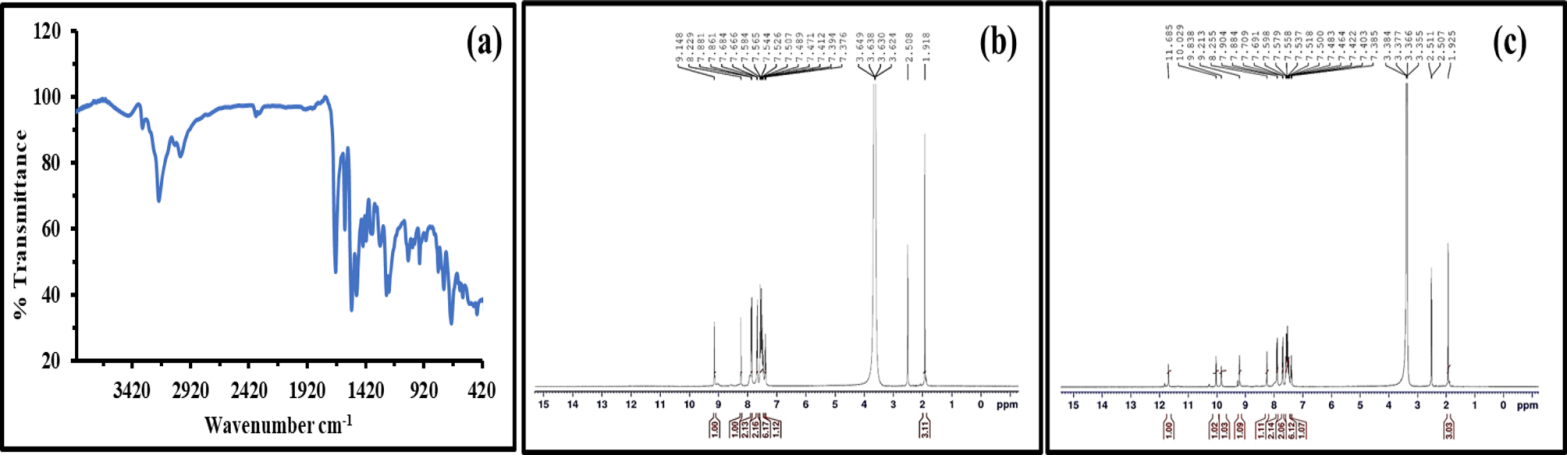
Spectral data of *PTH*: (**a**) FT-IR, (**b**) ^1^H NMR (DMSO-*d*_6_), and (**c**) ^1^H NMR (DMSO + D_2_O).

**Scheme 1:**
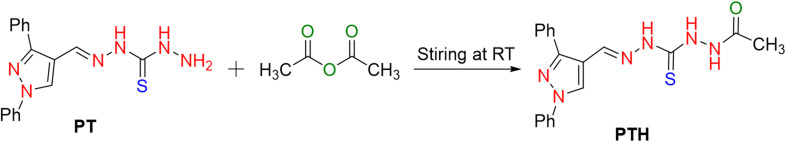
Synthesis of **PTH**.

Acetylation of compound **PT**, namely, *(E)-N’-((1*,*3-diphenyl-1 H-pyrazol-4-yl)methylene)hydrazinecarbothiohydrazide*^27^ was carried out as depicted in Scheme 1 using acetic anhydride by stirring at an ambient temperature for 4 h followed by filtration and recrystallization using ethanol to give beige crystals of **PTH** namely, *(E)-N’-(2-((1*,*3-diphenyl-1 H-pyrazol-4-yl)methylene)hydrazine-1-carbonothioyl)acetohydrazide* with a yield of 83%, and melting point (Mp = 210–212 °C). The *IR* spectrum of *PTH* using a Fourier-Transform Infrared Thermo Electron Nicolet iS10 Spectrometer in Fig. 1a displayed absorption bands for carbonyl functionality at ν 1678 cm^− 1^, NH groups at 3332 and 3193 cm^− 1^, and C = S group at 1243 cm^− 1^. The ^*1*^* H NMR* spectrum (δ, ppm) using AVANCE III 400 *MHz* spectrometer,

Bruker, Billerica, MA, USA), as depicted in Fig. 1b, c, offered signals at 1.92 (s, 3 H, CH_3_), 7.40 (t, 1 H, Ar-H, *J* = 7.6 *Hz*), 7.48–7.59 (m, 5 H, Ar-H), 7.69 (d, 2 H, Ar-H, *J* = 7.2 *Hz*), 7.89 (d, 2 H, Ar-H, *J* = 8.0 *Hz*), 8.25 (s, 1 H, CH = N), 9.21 (s, 1 H, C5-H pyrazole), 9.84 (*br*.s, 1 H, *NH*CO), 10.03 (*br*.s, 1 H, NHCS*NH*), 11.69 (*br*.s, 1 H, *NH*CSNH). The elemental analysis was performed on a Perkin-Elmer 2400 *CHN* elemental analyzer (Perkin-Elmer, Waltham, MA) reflecting C_19_H_18_N_6_OS (378.45): C, 60.30; H, 4.79; N, 22.21; Found: C, 60.21; H, 4.74; N, 22.23%.

### Carbon steel structure and electrolytes

The *CS* chemical composition (*%wt*) is listed in Table 1. *CS* surface underwent sequential polishing with various emery papers (400–2500) to achieve a mirror-like finish, followed by degreasing with acetone and rinsing with high-purity deionized water prior to each experimental procedure. A primary stock solution (1 × 10⁻² M) of the synthesized *PTH* in 1.0 M HCl was prepared with subsequent serial dilutions (5 × 10⁻⁴ − 5 × 10⁻⁶ M) carried out at room temperature (25 ± 1 °C).

**Table 1:** Chemical composition of *CS* (%wt).

Element	Fe	C	Si	Mn	Cr	Ni	*P*	S	O	*N*
**%wt**	94.00	0.0	0.60	1.00	2.00	1.20	0.03	0.02	0.85	0.40

### Corrosion measurements

The electrochemical characteristics of *CS* in aggressive HCl solution, both in the absence and presence of varying concentrations of *PTH* inhibitor, were examined through gravimetric analysis (weight loss, *WL*) and electrochemical methods (*EMs*), including electrochemical impedance spectroscopy (*EIS*) and potentiodynamic polarization (*PDP*) techniques. *EMs* were carried out using Potentiostat/Galvanostat (Origalys), with a platinum wire functioning as the auxiliary electrode (*AE*), Ag/AgCl (3 M KCl) serving as the reference electrode (*RE*), and *CS* acting as the working electrode (*WE*). These electrochemical evaluations were conducted following a 30-minute open-circuit potential (*OCP*) stabilization period, both with and without the *PTH* inhibitor at various concentrations. *EIS* was performed over a frequency range of 100 kHz to 0.1 *Hz*, utilizing an amplitude of 5 mV, which facilitated the determination of several kinetic parameters, including solution resistance (*Rs*), polarization resistance (*R*_*P*_), and constant phase element (*CPE*). Subsequent to *EIS*, *PDP* measurements were executed at a scan rate of 1.0 mV/s across a potential range of ± 300 mV centered around the *OCP*. From the Tafel plots, parameters such as corrosion current density (*i*_*corr*_), corrosion potential (*E*_*corr*_), anodic and cathodic Tafel slopes (*β*_*a*_ and *β*_*c*_), along with the corrosion rate (*r*), were derived and analyzed. Additionally, the effectiveness of the *PTH* inhibitor at a concentration of 5 × 10⁻⁴ M was evaluated and discussed through *EMs* at varying temperatures (25, 30, 40, and 50 °C) and over different time durations. Each experiment was performed 3 times at each concentration; also, the uncertainty values for the polarization resistance (*R*_P_) and those of the corrosion current.

densities (*i*_corr_) have been calculated.

### Theoretical quantum chemical study

Theoretical quantum studies regarding the analyzed *PTH* inhibitor were performed using BIOVIA Materials Studio 6.0 (17.1.0.48) software, https://www.3ds.com/products-services/biovia/products/molecular-modeling-simulation/biovia-materials-studio. The geometry optimization of *PTH* was carried out through *DFT* with the DMol3 module, which employed the GGA (generalized gradient approximation) method and utilized the DNP-3.5 (Double Numerical plus polarization) basis set to provide insights into the structural reactivity of the investigated *PTH*. Additionally, a theoretical assessment of the adsorption properties of *PTH* on Fe (110) surface was conducted in both gas and aqueous phases through *MCs* using the adsorption locator module, which is governed by the COMPASS (Condensed phase Optimized Molecular Potentials for Atomistic Simulation Studies) forcefield, to evaluate the effectiveness of *PTH* in reducing Fe corrosion in a 1.0 M HCl solution.

### Surface morphology examination

The corrosion behavior of *CS* and the protective efficiency of *PTH* were studied using *SEM* via QUANTA FEG 250-SEM after 10 h of immersion using an accelerating voltage of 20 keV. To further confirm the inhibitory action of *PTH*, *EDX* integrated with *SEM* was utilized to determine the elemental composition of the corroded and protected surfaces, which was also confirmed employing *AFM* using Park System XE-100 AFM. Additionally, *XPS* was applied as a high-resolution quantitative technique to analyze the metal/solution interface using a KRATOS XSAM-800.

## Results and discussion

### Weight loss (WL) measurements

In the gravimetric analysis, *CS* coupons with dimensions (1.0 × 1.0 × 0.7 cm) were meticulously polished to a mirror-like finish, then rinsed with deionized water and acetone to eliminate residual contaminants before being introduced into the test solution. The corrosion rate (*r*), inhibition efficiency (*η%*), and the surface coverage fraction (*θ*) by *PTH* molecules were determined using the following Eqs^2,28^. :

1 $$\:r=\:\frac{{W}_{o\:}-W}{A\times\:t\:}$$

2 $$\:\theta\:=\:\frac{{W}_{o\:}-W}{{W}_{o}}\:$$

3 $$\:\eta\:{\%}=\theta\:\times\:100\:\:\:\:$$

**Fig. 2:**
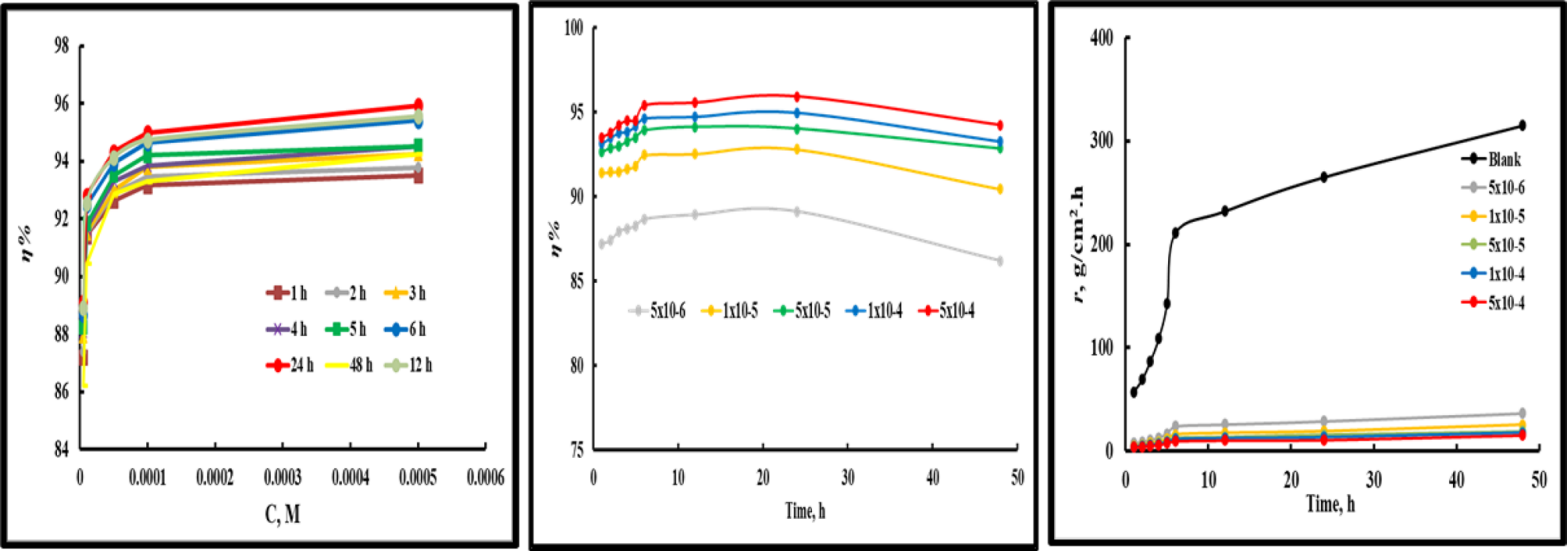
The variation of *r* of *CS* and *η* values of the studied *PTH* with time.

Where *A* is the exposed *CS* surface area (cm^2^), *r* is the corrosion rate (g/cm^2^ h), t means immersion time (h), and *W* and *W*_*0*_ in grams (g) denote the *WL* values with and without *PTH* addition, respectively^29^. Gravimetric analysis was conducted in 1.0 M HCl solution to evaluate *r* values under varying exposure durations and different concentrations of the investigated *PTH*, as shown in Fig. 2. Table 2 demonstrates that *PTH* acted as an effective inhibitor with $\:\eta\:\%$ increased with its concentration till it reached 93.51% and 95.93% after 1 h and 24 h, respectively at 5 × 10⁻⁴ M. This fact indicated the protective power of *PTH* against the destructive effect of HCl, which can be explained by *PTH* adsorption on the *CS* surface and generating a protective layer^18,19,30^. As noted, the mitigation potency increased with time, which reflected the strong inhibition performance of the studied *PTH*. Additionally, after a long immersion time of 48 h, the corrosion efficacy declined slightly, which can be attributed to the aggressive attack of the acidic environment, causing defects or pores in the inhibitor film. Also, Table 2 exhibited that *r* value increased slightly with *PTH* addition over time, reaching 15.24 g/cm^2^ h compared with the *r* value of the untreated solution, 315 g/cm^2^ h after 48 h at 5 × 10⁻⁴ M. As noticed, the addition of the studied inhibitor shifted the corrosion rate to lower values as concentration rising accompanied with enhancement in the mitigation efficiency. The decline in *r* values exhibited that *CS* was protected effectively by the introducing *PTH* which can be clarified by its high adsorption capacity over *CS* surface via the active centers in its molecular structure. As observed, in the presence of *PTH*, *r* values were significantly low compared with that in free HCl solution reflected the adsorption of *PTH* molecules over *CS* surface and formation of a barrier layer against the aggressive action of HCl solutions. Also, the enhancement in *r* values with time can be explained by some defects in *PTH* film caused by the aggressive HCl which evolved H_2_ gas. All these annotations reflected the protected film stability of *PTH* against the corrosive agents via its adsorption process over the *CS* surface *via* hetero atoms (N, O, and S), C = N, and aromatic rings in the *PTH* molecular structure^31–33^.

**Table 2:** *WL* measurements of *CS* in 1 M HCl solution and after the addition of *PTH* inhibitor.

Conc. (M)	1 h		2 h		3 h		4 h		5 h		6 h		12 h		24 h		48 h	
	*r* x10^− 4^ (g/cm^2^.hr)	η%	*r* x10^− 4^ (g/cm^2^.hr)	η%	*r* x10^− 4^ (g/cm^2^.hr)	η%	*r* x10^− 4^ (g/cm^2^.hr)	η%	*r* x10^− 4^ (g/cm^2^.hr)	η%	*r* x10^− 4^ (g/cm^2^.hr)	η%	*r* x10^− 4^ (g/cm^2^.hr)	η%	*r* x10^− 4^ (g/cm^2^.hr)	η%	*r* x10^− 4^ (g/cm^2^.hr)	η%
Blank	57	ـــــــــ	69	ـــــــــ	87	ـــــــــ	109	ـــــــــ	143	ـــــــــ	211	ـــــــــ	232	ـــــــــ	265	ـــــــــ	315	ـــــــــ
5 × 10 ^−6^	7.3	87.2	8.7	87.4	10.5	87.93	13	88.1	16.8	88.25	23.9	88.67	25.7	88.92	28.82	89.12	42.52	86.51
1 × 10 ^−5^	4.9	91.4	5.9	91.45	7.4	91.5	9.1	91.65	11.7	91.82	15.9	92.46	17.3	92.54	19.03	92.81	30.31	90.37
5 × 10 ^− 5^	4.2	92.63	4.9	92.9	6.1	92.98	7.3	93.3	9.3	93.5	12.7	93.93	13.6	94.13	15.01	94.33	22.92	92.72
1 × 10 ^− 4^	3.9	93.16	4.5	93.48	5.4	93.79	6.7	93.85	8.3	94.2	11.3	94.64	12.2	94.74	13.29	94.98	19.86	93.69
5 × 10 ^− 4^	3.7	93.51	4.3	93.77	5	94.25	6	94.5	7.9	94.51	9.7	95.4	10.3	95.56	10.78	95.93	16.94	94.62

### Electrochemical impedance spectroscopy

**Fig. 3:**
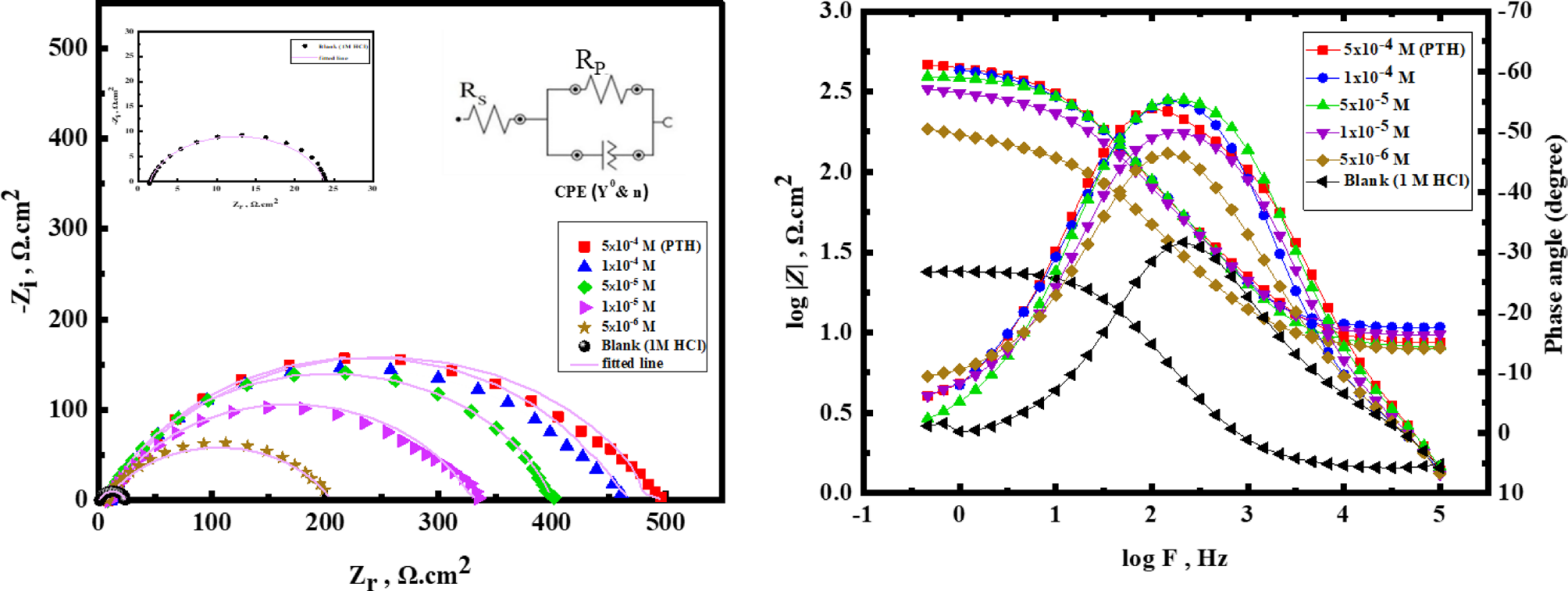
Nyquist plots for *CS* in 1 M HCl in the absence and presence of various concentrations of *PTH* at room temperature using the proposed equivalent circuit.

The Nyquist and Bode-phase plots in Fig. 3 provide detailed insights into the corrosion behavior of CS in an aggressive acidic HCl solution at room temperature, with and without the addition of *PTH* doses. In the absence of *PTH*, the Nyquist diagram displayed a small semicircular arc, indicating a low charge transfer resistance accompanied by a high corrosion rate^34^. However, with *PTH* concentration rising, the diameter of the Nyquist semicircle expands significantly, which can be elucidated by the enhanced *PTH* adsorption onto the *CS*, which improved its surface coverage, forming a protective layer that significantly impedes the electrochemical reactions occurring at the metal/solution interface^35^. The augmentation in Nyquist diameter directly correlates with the charge transfer resistance. At higher concentrations, *PTH* likely forms a more adhesive and uniform adsorption layer, shielding the *CS* surface from the aggressive media^36–38^. Bode-phase plots with a rise in phase angle towards 90° due to the relaxation effect induced by the adsorption of *PTH* molecules and Bode curves to higher values, confirming the protective effect of *PTH*.

Also, no significant alteration in the overall corrosion mechanism was observed after *PTH* addition, exhibiting that the primary corrosion process remains dominated by the charge transfer process^39^. Additionally, the compressed semicircular shape of the Nyquist diagrams in Fig. 3 reflects surface roughness and inhomogeneities on *CS*^40,41^. At low frequencies, the charge transfer resistance value showed a marked increase with the addition of *PTH* relative to that value in its absence, indicating that the adsorption of *PTH* molecules on the *CS* surface enhanced the covering insulation film formation that obstructed metal/HCl contact^42,43^.

As depicted in Fig. 3, the Bode modulus at lower frequencies enlarged with *PTH* concentration rising to higher values owing to adsorption enhancement and surface coverage of *CS* with *PTH* molecules^44^. This phenomenon confirmed the effective protection power of the studied *PTH* for *CS* by construction of a stable defensive film that mitigated the corrosion process in aggressive HCl solution^45^. The proposed equivalent circuit is shown in Fig. 3 includes the solution resistance (*R*_*s*_), polarization resistance (*R*_*P*_ = *R*_ct_ (charge transfer resistance) + *R*_d_ (diffuse layer resistance), *R*_a_ (accumulations at metal/solution resistance), and *R*_f_ (film resistance), and a constant phase element (*CPE*). The obtained values presented in Table 3 indicate that *CPE* is characterized by parameters *Y*^*o*^ and *n*. The n value dropped with the introduction of *PTH*, suggesting a decline in *CS* surface heterogeneity due to the adsorption of *PTH* at the *CS*/solution interface. Additionally, *Y*^*o*^ value decreases with the introduction of *PTH*, which reflects that the film thickness formed over the *CS* surface increased^39,46,47^. The next equation was applied for *Z*_*CPE*_ calculation:

**Table 3:** *EIS* parameters for *CS* in 1 M HCl solution with and without different concentrations of *PTH* inhibitor at room temperature.

Inh.	Conc. M	*R*_s_ (Ω.cm^2^)	*R*_*P*,_ (Ω.cm^2^)		Y^O^, µsn Ω ^−1^ cm^2^	*n*	C_dl_ (F/cm^2^)	F, Hz × 10^− 5^	t, s	θ	η%
			value	±uncertainty							
**Blank**	**0.00**	1.463	22.26	1.663921	0.372 6e-3	0.885	9.02	79.3	4.47E-03	**ـــــــــــ**	**ـــــــــــ**
***PTH***	**5 × 10** ^− 6^	7.235	197.9	1.153256	0.281 8e-3	0.738	1.26	63.89	2.01E-03	0.8875	88.75
	**1 × 10** ^− 5^	9.181	320.4	1.850225	9.72E-05	0.745	1.12	44.44	9.52E-03	0.9305	93.05
	**5 × 10** ^− 5^	8.032	392.6	2.36646	6.48E-05	0.790	1.01	40	9.61E-03	0.9433	94.33
	**1 × 10** ^− 4^	10.30	448	1.205543	7.33E-05	0.782	0.78	44.44	1.27E-02	0.9503	95.03
	5 × 10^− 4^	8.279	481.7	1.761628	9.11E-05	0.741	0.66	50	1.47E-02	0.9538	95.38

4 $$\:{Z}_{CPE}={Q}^{-1}(i{{\upomega\:}}_{max}{)}^{-n}$$

Here, *Q* and *ω*_*max*_ represent the constant phase element and the angular frequency, respectively^48,49^. According to the data in Table 3, a noticeable enhancement in the values of *R*_*s*_ was detected with *PTH* addition, suggesting a decrease in the solution conductivity, which indicated that the *CS* surface became progressively shielded from the corrosive environment^47,50,51^. A significant increase in *R*_***P***_ was observed with values reaching 197.9 Ω.cm² and 481.7 Ω.cm² for *PTH* concentrations of 5 × 10^−6^ M and 5 × 10^−4^ M, respectively, relative to the *R*_***P***_ value for the free HCl solution of 22.26 Ω·cm². This marked increase indicates a strong adsorption of *PTH* molecules on the *CS* surface, which effectively enhances protection against the aggressive environment^20,52^. To quantify the extent of corrosion inhibition, *θ* and *η* were derived from the *R*_***P***_ values using the following equation:

5 $$\:\theta\:=({R}_{P.\:\:PTH}-{R}_{P.\:\:1\:M\:HCl})/{R}_{P.\:\:PTH})$$

6 $$\:\eta\:{\%}=\theta\:\times\:100$$

*η%* showed a clear rising trend with increasing *PTH* concentration, reaching 88.75% and 95.38% at 5 × 10^− 6^ M and 5 × 10^− 4^ M, respectively. This demonstrated that *PTH* effectively retarded the corrosion process of *CS*, with the efficiency improving as more *PTH* molecules adsorbed onto the metal surface, forming a defensive layer limiting the exposure of the *CS* surface to the corrosive species through a displacement process with *PTH* molecules which enhanced film thickness (*T*) of the adsorbed layer. Subsequently, declined double-layer capacitance (*C*_*dl​*_)^53–55^. The *C*_*dl*_ and relaxation time (*τ*) can be computed from the following equations:

7 $$\:{C}_{\text{d}\text{l}}=\left(\frac{{\epsilon\:}^{^\circ\:}\epsilon\:}{T}\right)A$$

8 $$\:{C}_{\text{d}\text{l}}=1/\left(2{\uppi\:}{R}_{ct}{F}_{img\to\:Max}\right)$$

9 $$\:{\uptau\:}={C}_{\text{d}\text{l}}\times\:{R}_{\text{c}\text{t}}$$

Here, *A*, *ε*, and *ε°* denote *CS* surface area, permittivity of the local dielectric medium, and dielectric constant of air, respectively^40,41^. In addition, the introduction of *PTH* leads to a significant increase in *τ*, with a value of 0.0147 s at 5 × 10⁻⁴ M relative to the untreated solution of 0.00447 s. This suggests that the slow *PTH* adsorption onto the *CS* surface, and the adsorption time process becomes much higher with the formation of a more stable and robust protective film via several active sites as aromatic rings, hetero atoms (*N*,* S*, and *O*), and π-electrons, consequently decreasing *CS*/HCl contact^56,57^.

### Potentiodynamic polarization (PDP)

**Fig. 4:**
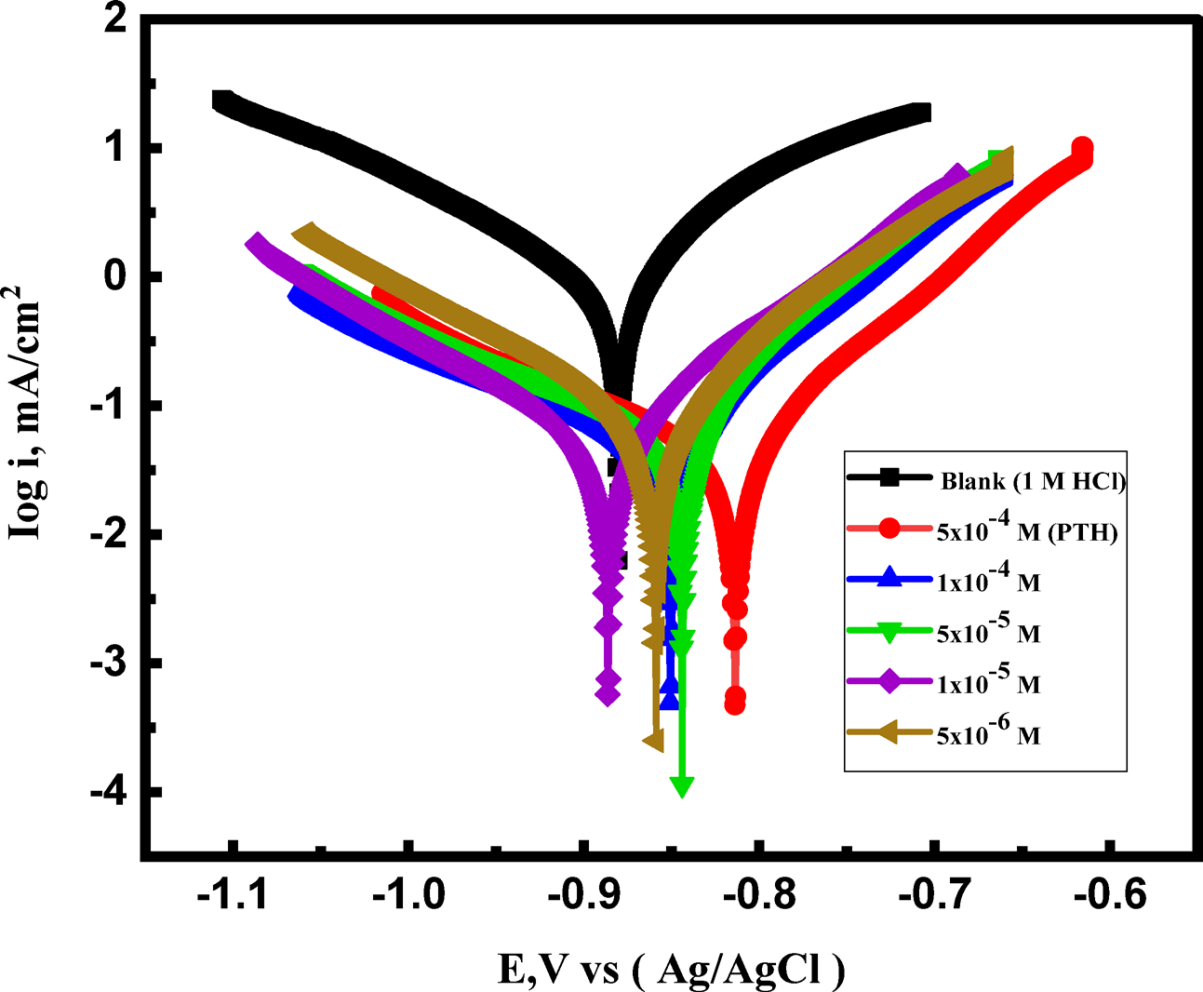
*PDP* curves of *CS* in 1 M HCl in the absence and presence of various concentrations of *PTH* inhibitor at room temperature.

The mitigation performance of the *PTH* for *CS* corrosion in the HCl environment was assessed using the *PDP* technique. The polarization curves revealed a significant shift in both the anodic and cathodic Tafel lines in Fig. 4 towards less corrosion current densities upon the addition of *PTH*, indicating a strong adsorption of the inhibitor molecules on the *CS* surface, blocking its active sites, thereby hindering the dissolution process and reducing the corrosion rate^58,59^. As illustrated in Fig. 4, the difference observed between log *i* versus *E* curves for the *PTH* inhibitor and the blank solution, which was augmented with *PTH* concentration, confirmed *PTH* adsorption onto the *CS* surface. This observation suggested that the presence of *PTH* markedly reduced the corrosion process of *CS*^34^.

In the cathodic region, the parallelism in Tafel curves reflected the ability of *PTH* to reduce the hydrogen evolution rate by shielding the *CS* surface from H⁺ ions, which bolstered that the evolution of H_2_ was activated and controlled without altering the corrosion mechanism^60,61^. At the anodic region, the investigated *PTH* inhibited the *CS* dissolution reaction effectively with a change in anodic line slope, accompanied by a decline in the corrosion current density, which demonstrated the protective film construction through *PTH* adsorption, limiting the contact between the corrosive medium and *CS*^62^. *PDP* parameters derived from Tafel extrapolation (Table 4) confirmed that the injection of *PTH* reduced the *i*_*corr*_ significantly till reach 26.47 µA cm⁻² at 5 × 10⁻⁴ M, relative to that value in the uninhibited solution of 627.051 µA cm⁻². This reduction correlated with a rise in the mitigation competence, indicating the mitigation power of the studied *PTH*^63^. The values of *η* and *θ* were calculated using the following Eqs^56,64^.

**Table 4:** *PDP* parameters for *CS* in 1 M HCl solution with and without different concentrations of *PTH* inhibitor at room temperature.

Inh.	Conc. (M)	E_corr_, (mv)vs. Ag/AgCl)	β_a_ mV/dec)	-β_c_ (mV/dec)	i_corr,_ µA/cm²		*r* (mm/year)	*R*_p_ (Ω.cm^2^)	θ	η%
					value	±uncertainty				
**Blank**	**0.00**	−882.30	60.9	108.8	627.05	1.809926	14.73	27.1	**ـــــــــــ**	**ـــــــــــ**
	**5 × 10** ^− 6^	−858.61	53.9	89.7	65.04	2.116727	1.527	225	0.8963	89.63
	**1 × 10** ^− 5^	−885.72	52.6	83.4	41.55	1.900772	0.976	337	0.9337	93.37
***PTH***	**5 × 10** ^− 5^	−847.77	43.6	96.6	35.08	2.103838	0.824	372	0.9441	94.41
	**1 × 10** ^− 4^	−853.01	49.5	91.1	30.72	2.830848	0.721	454	0.9510	95.10
	**5 × 10** ^− 4^	−815.58	44.3	98.8	26.47	1.664342	0.621	485	0.9578	95.78

10 $$\:\theta\:=({i}_{\text{c}\text{o}\text{r}\text{r}.\:\text{H}\text{C}\text{l}}-{i}_{\text{c}\text{o}\text{r}\text{r}.\:\:PTH})/{i}_{\text{c}\text{o}\text{r}\text{r}.\:\text{H}\text{C}\text{l}}$$

11 $$\:\eta\:\%=\theta\:\:\times\:\:100$$

where:

- *i*_corr−blank_ is the corrosion current density of the uninhibited solution.
- *i*_corr−inh_ is the corrosion current density after the addition of *PTH*.

*PTH* addition to the acidic medium, significantly diminished both the cathodic (H₂ evolution) and anodic reactions, demonstrating its effective inhibition. After the injection of *the PTH* inhibitor, the corrosive medium gains more anodic potentials, especially at higher concentrations, and both the anodic and cathodic current densities decrease. So, it could be concluded that the *PTH* acted as a mixed-type corrosion inhibitor. The investigated *PTH*, in particular, slows both the rate of anodic metal dissolution and the rate of cathodic hydrogen gas evolution. Moreover, the variation in *E*_corr​_ was within a ± 85 mV range relative to the untreated solution, supporting the mixed-type inhibition of *PTH via* hindering both anodic and cathodic reactions of^30,65,66^ Also, the *β*_*c*_ values in Table 4 are very close together, implying that the *PTH* inhibitor reflected that no change in the cathodic hydrogen evolution reaction which can be clarified by the adsorption of *PTH* molecules onto *CS* surface decreasing the available surface area for H^2^ production exhibiting that the cathodic reduction reaction can only take place on the uncovered *CS* surface or if the H^+^ ions reach the *CS* surface through the pores of the inhibitor film.

On the other hand, the existence of *PTH* suppressed the rate of anodic current density and *β*_*a*_ by almost 30% which reveals that the injection of *PTH* inhibitor alters the mechanism of the anodic reaction and prevents it from corrosion^31,67,68^. Additionally, as *PTH* doses increased, the inhibition ability also increased, owing to the rise of *PTH* adsorption capacity over the *CS* surface, accompanied by extra surface coverage of its active sites^63^. The studied *PTH* protected the *CS* surface with mitigation competence 95.78% at 5 × 10⁻⁴ M, which can be attributed to shielding of more exposed *CS* surface area via replacement of the adsorbed corrosive particles by the adsorbed *PTH* molecules, forming a stronger protective barrier^42^. All these findings from *PDP* and *EIS* techniques reinforced the inhibitory power of the prepared *PTH* and its ability to protect *CS* in the aggressive acidic environment.

### Adsorption performance and film stability

The stability of *PTH* film on *CS* surface in 1.0 M HCl at 5 × 10⁻⁴ M was evaluated under extreme conditions, including varying temperatures and extended immersion periods, using the *EIS* technique.

- ***Temperature effect***.

**Fig. 5:**
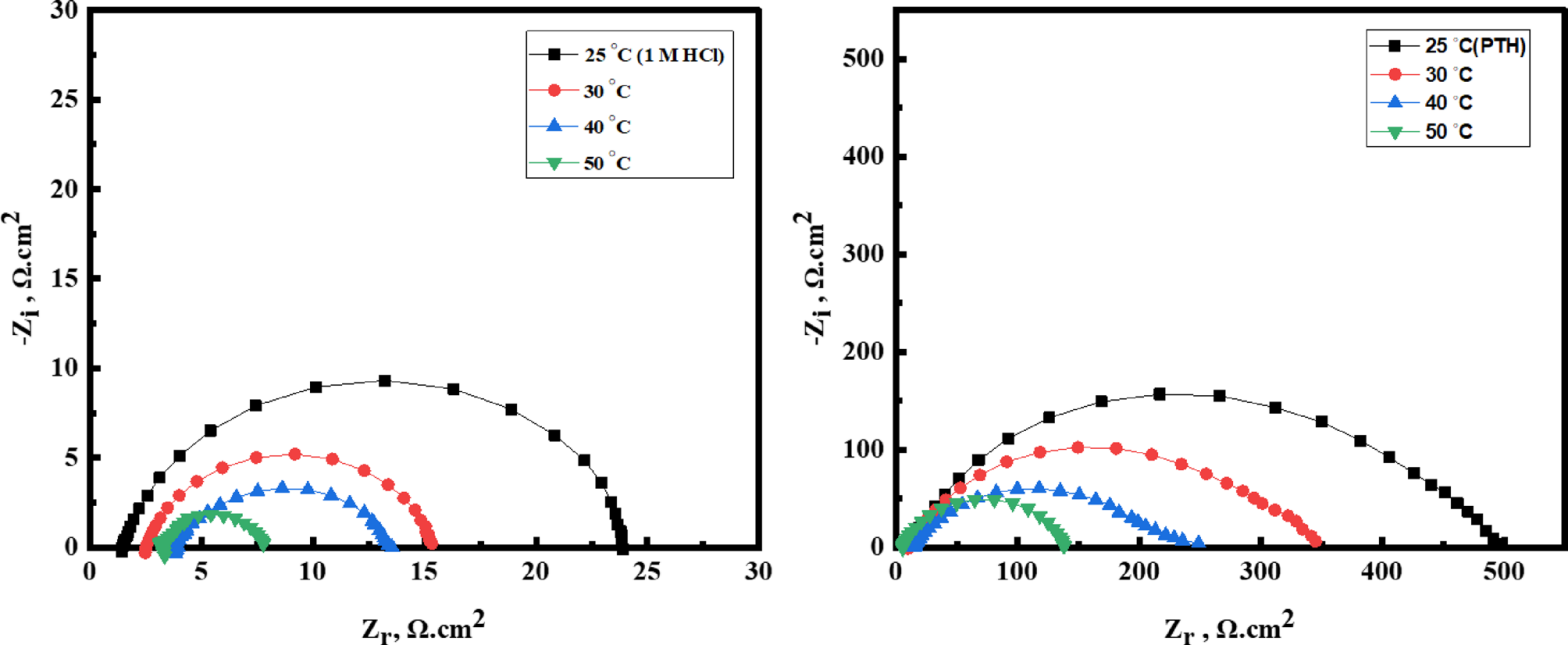
Nyquist curves for *CS* in 1 M HCl in the absence and presence of 5 × 10^− 4^ M of *PTH* inhibitor at different temperatures.

The effectiveness of the *PTH* inhibitor in controlling *CS* corrosion in a 1.0 M HCl environment was evaluated over various temperature levels (25–50 °C). As the temperature increased, the system’s kinetic energy and the mobility of corrosive ions heightened, leading to a decrease in the stability of both the corrosion product layers and the defensive *PTH* film on the *CS* surface^69^. Figure 5 illustrates Nyquist plots of *CS* in 1.0 M HCl, with and without 5 × 10⁻⁴ M of *PTH*, displayed semicircular patterns with a diameter that declined with the temperature enhancement, suggesting that the corrosion mechanism remained consistent despite temperature fluctuations^70^. This fact indicated the protective role of *PTH* at higher temperatures by forming a stable and resilient adsorption layer on the *CS* surface, which reduced the adverse effect of the aggressive acidic environment^71,72^. Table 5 demonstrates a gradual increase in *η%* values with rising temperature, reaching 97.16% at 50 °C. This reflected the inhibition power of *PTH* for *CS* with temperature rising which can be explained by the attraction and adherence of the *PTH* molecules with *CS* surface foaming a stable film layer against the destructive action of HCl solution via chemical bonds between *PTH* active centers and vacant 3d-orbital of Fe^48,49^.

**Table 5:** *EIS* parameters for *CS* in 1 M HCl, free and containing 5 × 10^− 4^ M of *PTH* inhibitor at different temperatures.

Inh.	Temp. ^◦^C	*R*_s_, (Ω.cm^2^)	*R*_*P*_, (Ω.cm^2^)	θ	η%
**Blank**	25	1.463	22.26	----	----
	30	2.5	13.03	----	----
	40	3.879	9.386	----	----
	50	3.111	4.77	----	----
***PTH***	25	8.279	481.7	0.9538	95.38
	30	11.85	360	0.9638	96.38
	40	13.88	238.8	0.9601	96.01
	50	19.4	168	0.9716	97.16

- ***Immersion time effect***.

**Fig. 6:**
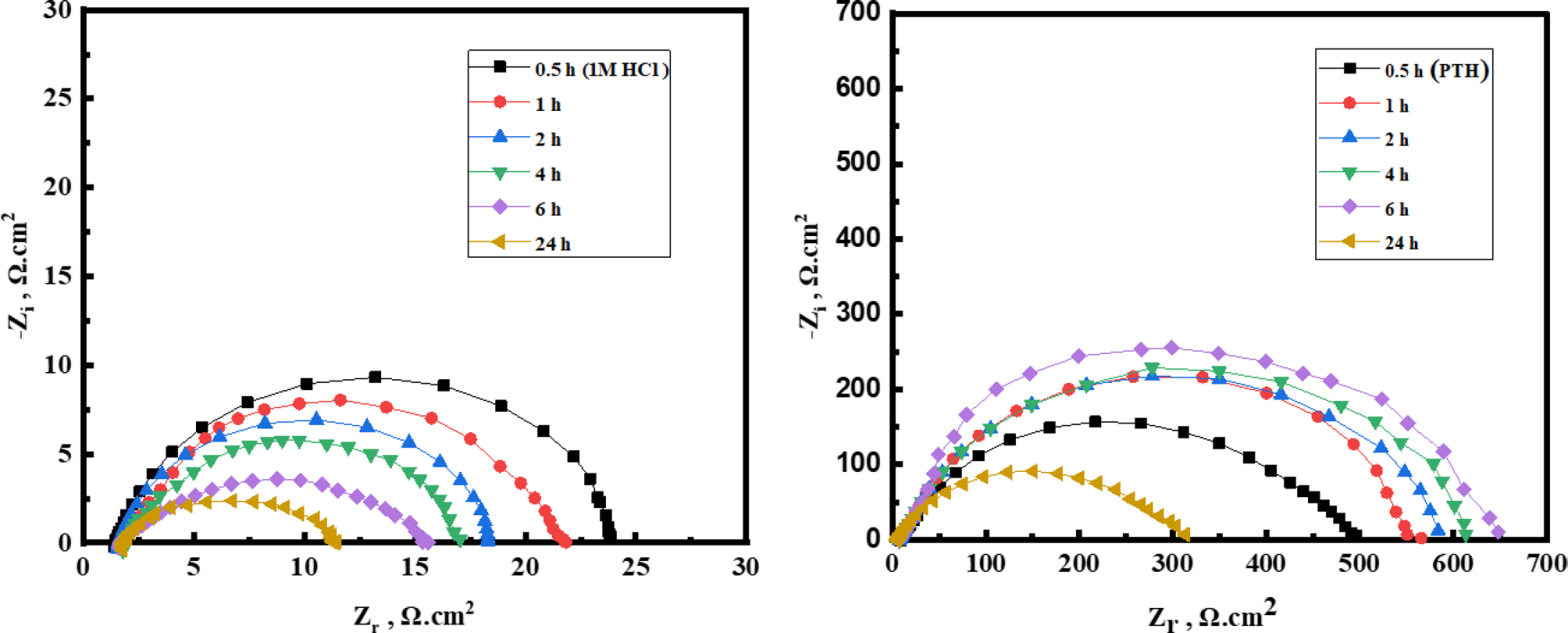
Nyquist curves for *CS* in 1 M HCl in the absence and presence of 5 × 10^− 4^ M of *PTH* inhibitor after different immersion times.

Moreover, long-term exposure studies could offer valuable insights into the temporal stability of the protective layer formed by *PTH*. Monitoring changes in the impedance spectra over time would help assess whether the protective film becomes more stable or desorbs from the metal surface. The exposed *CS* surface in 1.0 M HCl, free and containing 5 × 10⁻⁴ M of *PTH* across varying immersion periods as shown in Fig. 6. The *η%* value was monitored over time, as outlined in Table 6, reflecting its value exhibited a progressive increase till touch 97.3% after 24 h of immersion. This trend highlighted the stability of the *PTH* film over the *CS* surface with the gradual accumulation of *PTH* molecules, effectively reducing the corrosive impact of HCl^73,74^.

**Table 6:** *EIS* parameters for *CS* in 1 M HCl, free and containing 5 × 10^–4^ M of *PTH* inhibitor at different immersion times.

Inh.	t, h	*R*_s_, (Ω.cm^2^)	*R*_*P*,_ (Ω.cm^2^)	θ	η%
**Blank**	0.5	1.463	22.26	----	----
	1	3.792	18.2	----	----
	2	1.599	16.87	----	----
	4	3.472	15.65	----	----
	6	3.7	12.3	----	----
	24	3.408	7.908	----	----
***PTH***	0.5	8.279	481.7	0.9538	95.38
	1	7.847	557	0.9673	96.73
	2	7.766	569	0.9704	97.04
	4	7.775	577	0.9729	97.29
	6	7.939	581.9	0.979	97.9
	24	4.874	292.2	0.973	97.3

The Nyquist plots (depicted in Fig. 6) illustrate the electrochemical impedance response of *CS* in the absence and presence of 5 × 10⁻⁴ M *PTH* following extended exposure. The presence of *PTH* shifted the *R*_*P*_ values to higher values relative to those of the uninhibited 1.0 M HCl solution^41,75^. This observation underlines the protective capability of the *PTH* layer, which serves as a barrier against corrosive species^76,77^. As detailed in Table 6, the *R*_*P*_ ​ values for *CS* in the blank 1.0 M HCl solution diminished over time (decreasing from 22.26 Ω.cm² to 7.908 Ω.cm²), reflecting an accelerated corrosion process due to the aggressive action of HCl with the exposure time^63,78^. As noted in Table 6, the *R*_*P*_ value for the *PTH-inhibited* system remained markedly higher than that for the blank solution, providing robust evidence of the inhibitor’s sustained effectiveness in mitigating *CS* corrosion with various immersion times^78,79^.

### Activation thermodynamic parameters

**Fig. 7:**
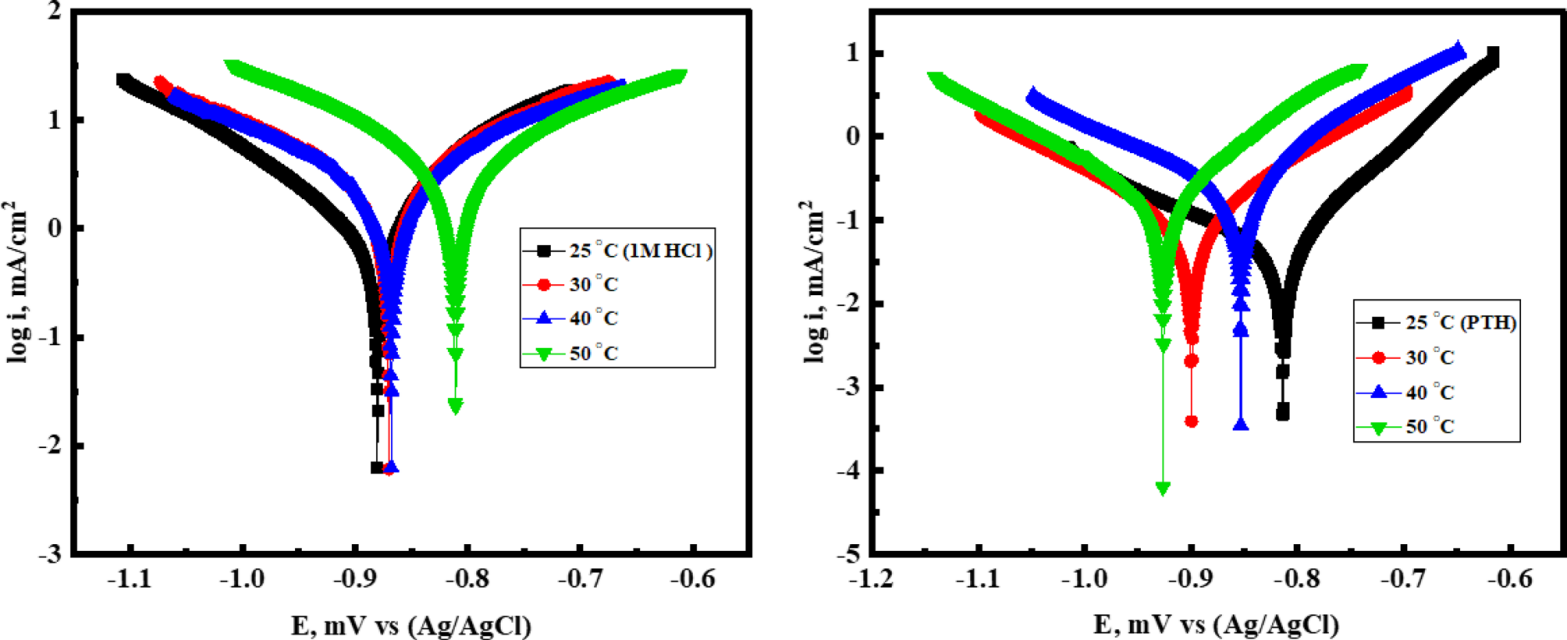
*PDP* curves of *CS* in 1 M HCl in the absence and presence of 5 × 10^− 4^ M of *PTH* inhibitor at different temperatures.

Potentiodynamic polarization (*PDP*) experiments were conducted at varying temperatures to assess the mitigation competence of the *PTH*. The semi-logarithmic *PDP* curves (Fig. 7) with their corresponding parameters were summarized in Table 7, which illustrated the electrochemical response of *CS* in both the uninhibited solution and after treatment with 5 × 10⁻⁴ M *PTH*. The data in Table 7 revealed a substantial decrease in *r* and *i*_*corr*_ values upon *PTH* addition compared to the uninhibited 1.0 M HCl solution, which confirms *PTH’s* strong anticorrosive efficacy owing to its adsorption onto the *CS* surface, facilitating the formation of a protective barrier layer^80–82^. Interestingly, the inhibition efficiency exhibited an increase from 95.78% at 25 °C to 96.2% at high temperature (50 °C), exhibiting the thermal stability of the protective film at elevated temperatures^83,84^. This fact suggested the formation of a robust and adherent shielding layer that resists HCl corrosive attack with a chemical adsorption mechanism of *PTH*^17^. Thermodynamic activation parameters, including activation energy (*E*_*a*_), activation entropy (ΔS*), and activation enthalpy (ΔH*), were calculated for both the uninhibited and *PTH-*treated systems based on r values utilizing Arrhenius and transition state equations as follows:

**Table 7:** *PDP* parameters for *CS* in 1 M HCl, free and containing 5 × 10^–4^ M of *PTH* inhibitor at different temperatures.

Inh.	Temp, °C	-E_corr_, (mV) Vs. Ag/AgCl	i_corr_ (mA/cm^2^)	β_a_ (mV/dec)	β_c_ (mV/dec)	*r*	*R*_*P*_	θ	η%
						(mm/year)	(Ω.cm2)		
**Blank**	25	882.30	627.05	60.9	108.8	14.73	27.1	-----	-----
	30	872.11	962.38	75.2	91.1	22.60	18.6	-----	-----
	40	870.83	1289.57	82.1	80.7	30.29	13.7	-----	-----
	50	810.92	1682.76	79.3	75.1	39.52	9.97	-----	-----
***PTH***	25	815.58	26.47	44.3	88.8	0.621	485	0.9578	95.78
	30	844.51	37.76	41.1	106.8	0.887	342	0.9608	96.08
	40	900.31	50.12	49.4	99.6	1.177	286	0.9611	96.11
	50	927.01	64.65	36	51.8	1.518	143	0.9620	96.20

12 $$\:\text{ln}r=\text{ln}\text{A}-\left(\frac{{E}_{a}}{RT}\right)$$

Arrhenius:

13 $$\:\text{ln}(r/\text{T})=\left[\text{ln}(\frac{\text{R}}{{N}_{A}h}\right)+\left(\frac{\varDelta\:{\text{S}}^{\text{*}}}{\text{R}}\right)]-(\varDelta\:{\text{H}}^{\text{*}}/\text{R}\text{T})$$

**Fig. 8:**
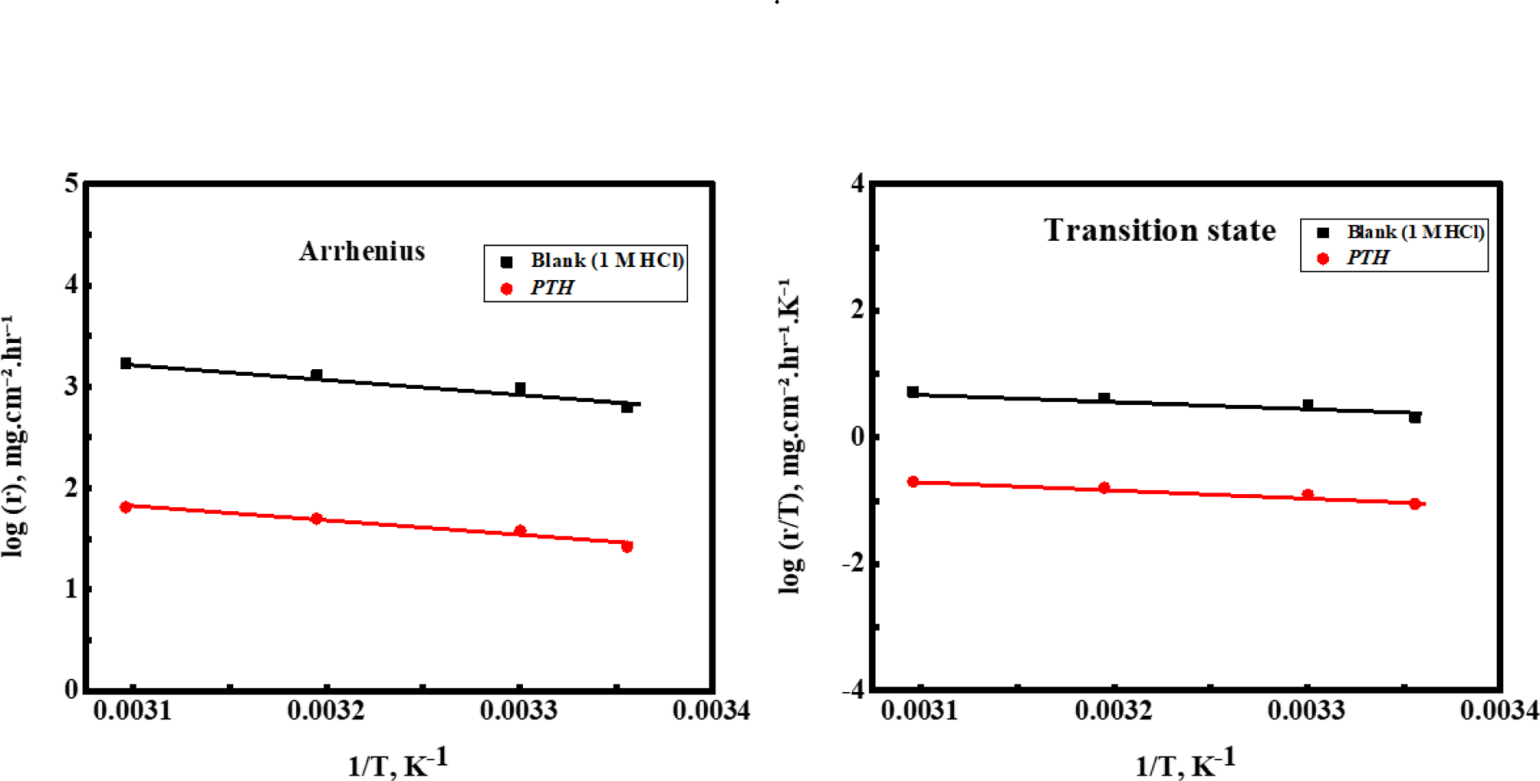
Arrhenius and transition state relations against 1/T for *CS* in 1 M HCl, free and containing 5 × 10 ^− 4^ M of *PTH*.

Transition state: 

Here, *A* is the Arrhenius constant, *T* denotes the absolute temperature, and *R* refers to the gas constant^85–87^. The corrosion behavior of *CS* is consistent with the Arrhenius equation, as depicted in Fig. 8, showing an observed linear relationship with a regression coefficient (*R²*) approaching unity^73,74^. *E*_*a*_ was determined and reported as in Table 8, with a drop in its value from 29.66 kJ/mol in 1.0 M HCl to 27.15 kJ/mol with *PTH* addition, indicating that *PTH* adsorbed chemically onto the *CS* surface^67,88–90^. The positive ΔH* value exhibited that the dissolution of *CS* was endothermic, which implies that the dissolution process becomes more difficult after the addition of the *PTH* inhibitor^84,91^. Additionally, the negative ΔS* value in Table 8 bolsters the formation of the activated complex during the rate-determining step over its dissociation, indicating a more ordered transition from reactants to the activated complex^81,82,92^.

**Table 8:** Activation thermodynamic parameters for *CS* in the absence and presence of *PTH* inhibitor at different temperatures.

Inh.	Arrhenius			Transition state			
	slope	*R* ^2^	E_a_ (KJ mol ^− 1^)	slope	intercept	ΔH* (kJ mol ^− 1^)	ΔS* (KJ mol ^− 1^)
**Blank**	−1549.2	0.9431	29.66	−14,145	5.1176	27.09	−99.59
***PTH***	−1417.7	0.96	27.15	−1283	3.2901	24.57	−134.59

### Adsorption isotherm

**Fig. 9:**
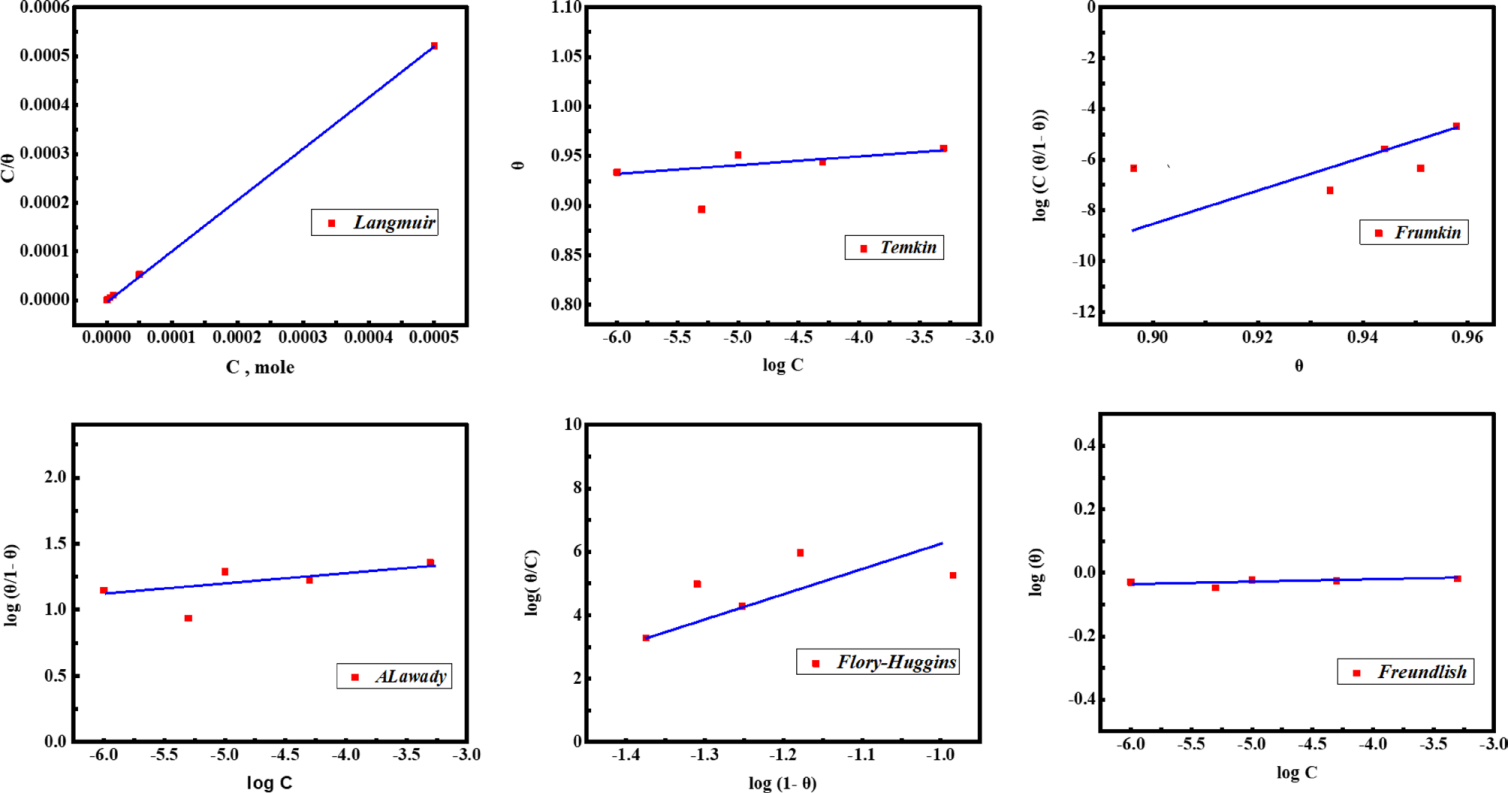
Various isotherms of *PTH* adsorption at the *CS*/HCl interface using *PDP* at room temperature.

Several adsorption isotherms were evaluated based on *θ* values obtained from *PDP*, as depicted in Fig. 9. Among the evaluated models, the Langmuir isotherm exhibited the best fit with a correlation coefficient (*R*^*2*^ = 1) using the following equation:

14 $$\:\raisebox{1ex}{$\text{C}$}\!\left/\:\!\raisebox{-1ex}{${\uptheta\:}$}\right.=\left(\frac{1}{{K}_{\text{a}\text{d}\text{s}}}\right)+\text{C}$$

Also, the standard Gibbs free energy of adsorption (*ΔG◦*_*ads*_​) was determined using the following equation:

15 $$\:\varDelta\:{G}_{ads}^{^\circ\:\:}=-\text{R}\text{T}\text{ln}\left(55.5{K}_{\text{a}\text{d}\text{s}}\right)$$

Here, *C* represents the *PTH* concentration (M), and *K*_***ads***_ ​ is the adsorption equilibrium constant. The constant 55.5 represents the molar concentration of water (mol/L)^30,93^. The *K*_*ads*_ values, as listed in Table 9, demonstrated a significant interaction between *PTH* and the *CS* surface through an electron-sharing (donor-acceptor) mechanism involving nitrogen atoms and the benzene ring with the unoccupied 3d-orbitals of Fe forming a stable chemisorbed *PTH* layer^4,94^. The adsorption process of the studied *PTH* can be clarified via various modes as physical adsorption through electrostatic attraction between the charged *PTH* centers.

**Table 9:** Langmuir isotherm parameters of *PTH* adsorption at the *CS*/HCl interface using *PDP* at room temperature.

Langmuir	Slope	intercept	*R* ^2^	K_ads_ L.mol^− 1^	ΔG^o^_ads_ kJ.mol^− 1^
***PTH***	1.0436	3.00E-07	1	3.33E + 06	−47.2

and the negative sites of the *CS* surface, and chemical adsorption *via* chemical bond (coordination) formation through electron sharing (donor–acceptor interactions) between.

heteroatoms (N, S, and O), and double bonds in C = N, C = O, C = S, and aromatic rings.

with *CS* vacant orbitals.

As the numerical value of $\:-\varDelta\:{G}_{ads}^{^\circ\:\:}$ is lower than 20, the physical adsorption reaction takes place. While the reverse (chemical) occurs when its value is higher than 40, and mixed type occurs when its value is in between them (20 *<* - $\:\varDelta\:{G}_{ads}^{^\circ\:\:}$*<* 40). The calculated $\:\varDelta\:{G}_{\text{a}\text{d}\text{s}}^{^\circ\:\:}$value was − 47.2 kJ/mol, confirming that *PTH* chemical adsorption over the *CS* surface^38,44^. In addition, the negative *ΔG◦*_*ads*_ value further indicated that the adsorption process was spontaneously characterized by a strong interaction and highly efficient surface coverage, and the examined *PTH* inhibitor offered the stability of the protective film at high temperature^95,96^. This behavior was also matched with other published literature.

### Computational approach

- **Density functional theory (*DFT*)**.

**Fig. 10:**
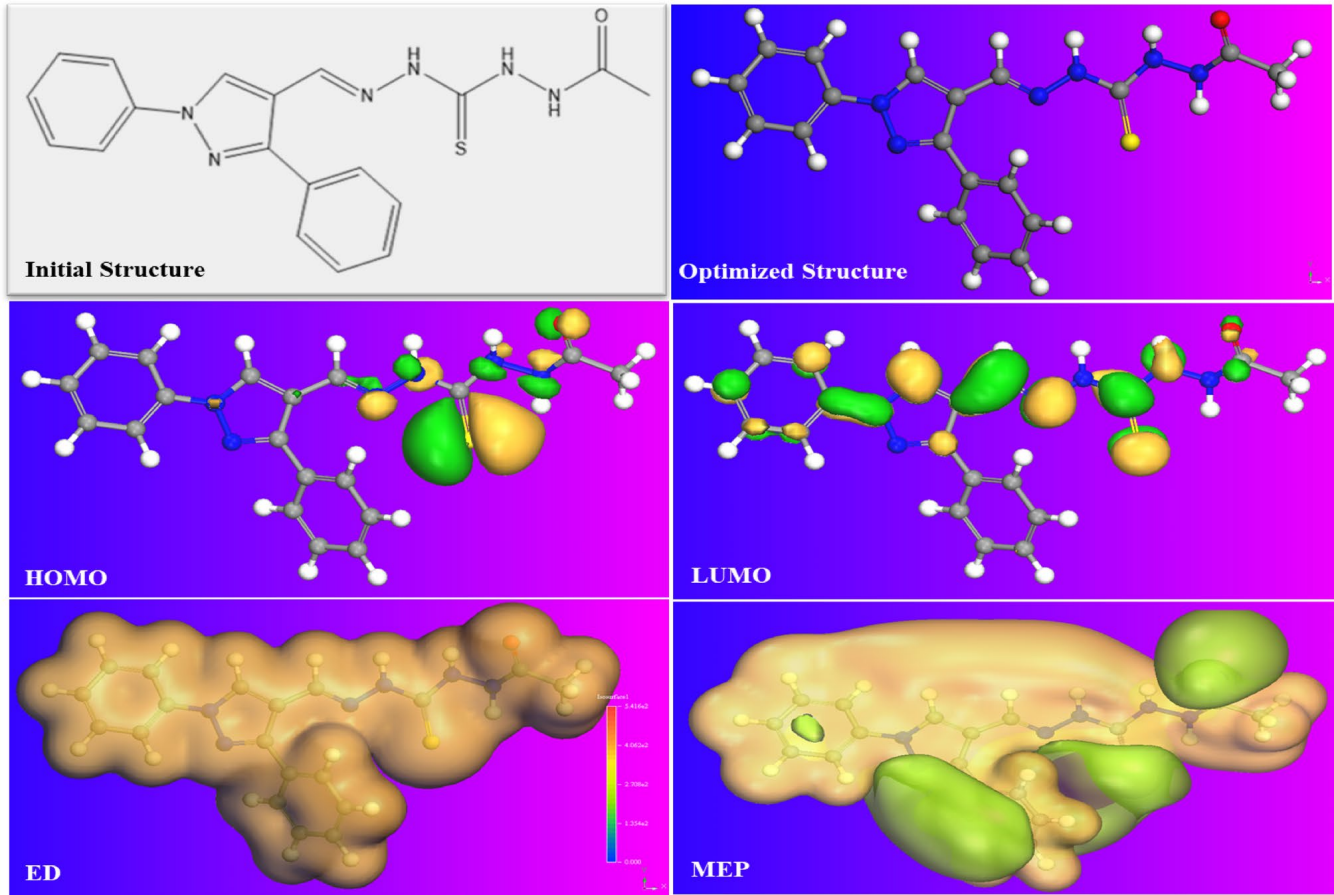
Optimized structure, *HOMO*, *LUMO*, *ED*, and *MEP* of the studied *PAS* inhibitor obtained by BIOVIA Materials Studio 6.0 (17.1.0.48).

Frontier molecular orbitals (*HOMO*: Highest Occupied Molecular Orbital and *LUMO*: Lowest Unoccupied Molecular Orbital) of the prepared *PTH* were illustrated as in Fig. 10, showing the *PTH* optimized structure characterized by the most stable arrangement of atoms with the lowest energy. The *PTH* planarity shape suggested the high likelihood of interaction of *PTH* with the *CS* surface^97–99^. *HOMO* and *LUMO* of *PTH* inhibitor reflected that *HOMO* corresponds to the electron cloud of nucleophilic centers distributed around hetero atoms such as the O-atom in the C = O group, the S-atom in the C = S group, and various N-atoms in the aliphatic chain. This configuration suggested the potential of chemical bond (coordination bond) formation *via* electron transfer to the empty d-orbital of Fe^60,61,69^. Conversely, *LUMO* represents the electron cloud of electrophilic centers localized around all hetero atoms (N, S, and O) in the *PTH* molecular structure and benzene and pyrazole rings, indicating the transfer of electron acceptance from the *CS* surface through the process of back donation^100,101^. The *HOMO* and *LUMO* were distributed throughout the entire *PTH* structure, indicating the electron sharing process between the *PTH* inhibitor and the *CS* surface. This observation was further validated by the Electron Density (*ED*) and molecular electrostatic potential (*MEP*) isosurfaces of *PTH* in Fig. 10. All these annotations confirm the adsorption capability of *PTH* upon the *CS* surface, leading to protective dense layer formation that shields the exposed *CS* surface against the corrosive ingredients^47,102,103^.

The energies of *HOMO* (*E*_*H*_) and *LUMO* (*E*_*L*_) were employed to derive the relative quantum indices, which are presented in Table 10. through the following equations:

**Table 10:** Quantum chemical parameters of the investigated *PTH.*

Comp.	E_H_ (eV)	E_L_ (eV)	ΔE_gap_ (eV)	η (eV)	E_b→d_ (eV)	χ (eV)	ΔN
*PTH*	−0.1761	−0.0764	0.0997	0.0498	−0.0124	0.12632	47.053

16 $$\:\varDelta\:E={E}_{L}-{E}_{H}$$

17 $$\:\eta\:=\frac{\varDelta\:E}{2}$$

18 $$\:\:\:\:\:\:\:\chi\:=\frac{-({E}_{HOMO}+{E}_{LUMO})}{2}$$

19 $$\:\varDelta\:N=\frac{({\phi\:}_{Fe}-{\chi\:}_{Inh})}{\left[2\right(\left({\eta\:}_{Fe}+{\eta\:}_{Inh.}\right)]}$$

20 $$\:\:{E}_{b-d}=-\left(\frac{{\eta}}{4}\right)$$

Here, Δ*E* represented the energy gap. *η* means global hardness, *E*_b→d_ refers to the energy of back donation. The parameters χ, Δ*N*, and φ corresponded to electronegativity, fraction of electron transfer, and work function of the Fe (110) plane (4.82 eV)^104,105^. *E*_*H*_ and *E*_*L*_ values indicate the interaction between the investigated *PTH* and *CS* surface through the electron sharing process (donation-acceptance)^83,106^. The energy gap (Δ*E*) is an index of reactivity or stability of the inhibitor molecule. As the value of Δ*E* is small as the electron polarizability is easy, and in turn, the higher adsorption efficiency of the tested molecule. The observed low Δ*E* value, as shown in Table 10, signifies enhanced electron polarizability; consequently, a greater adsorption capacity of *PTH* confirms its reactivity and chemical stability^60,66,107^. Also, *η* with a lower value exhibited a higher adsorption tendency of *PTH* upon the *CS* surface. Another important quantum descriptor that is used to confirm the relation between the inhibitor’s efficiency and its electron-donating ability to/or from the iron is the fraction number of electron transfer (*ΔN*). The high positive Δ*N* value of the studied *PTH* indicates its capacity to supply electrons to the unfilled Fe-3d orbitals.

So, the mitigation performance of the investigated *PTH* for iron corrosion increases with the increase in electron-exciting ability. Also, the negative sign of *E*_b→d_, indicated the electron transfer to the LUMO of *PTH* from the filled 3 d or/and 4 s orbitals of Fe. The donation and backdonation processes confirmed the adsorption capacity of the *PTH* inhibitor^106^. All these observations reinforced the defensive performance of the studied *PTH* for the *CS* surface immersed in acidic solution, that retards its corrosion rate through the adsorption process, and this is also in agreement with the published literature^20,31,107,108^.

- ***Monte Carlo simulation (MCs)***.

**Fig. 11:**
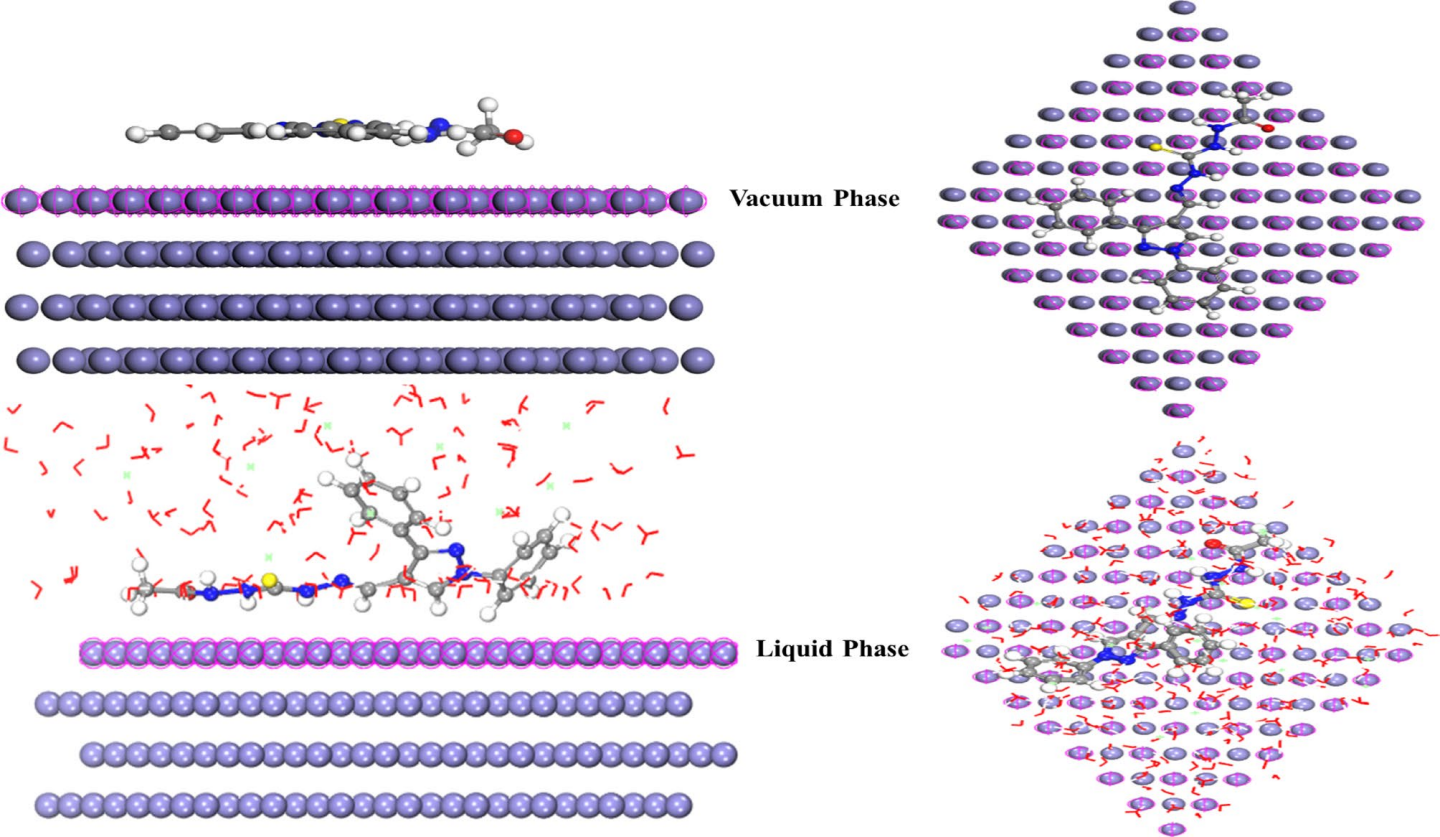
Equilibrium adsorption configuration of the studied *PAS* in both gas and liquid phases on Fe (110) obtained by MCs using BIOVIA Materials Studio 6.0 (17.1.0.48).

*MCs* serve as a strong approach for *PTH* adsorption onto Fe (110), as shown in Fig. 11 in both vacuum and aqueous phases. Figure 11 shows *PTH* with parallel orientation relative to the *CS* surface, which highlights the adsorption potential of *PTH* and the covering of extra *CS* surface area^83,109^. The parallel alignment of the studied *PTH* enabled the efficient interaction between *PTH* active sites and the Fe surface, providing substantial protection against HCl corrosive effects *via* electron sharing process (donor–acceptor interactions) between heteroatoms (N, S, and O), and double bonds in C = N, C = O, C = S, and aromatic rings.

in the *PTH* structure with Fe vacant 3d-orbitals^110,111^. Also, the orientation of the studied *PTH* active centers towards the Fe surface introduces a strong and stable insulation covering layer against the destructive agents. Energy of adsorption (*E*_*ads*_) is the energy released upon *PTH* inhibitor binding “relaxed” on Fe surface while the rigid adsorption energy (*E*_*rigid*_) is the released or required for the unrelaxed adsorbate (*PTH*) to be adsorbed on the adsorbent “Fe surface”, whereas the deformation energy (*E*_*def*_) is the released energy from the relaxation of the adsorbed *PTH* on Fe surface. The total Energy (*E*_*tot*_) is the sum of internal energy and *E*_*ads*_. The data derived from the *MCs* in Table 11 in both vacuum and aqueous phases clarified the adsorption capacity and strong interaction between *PTH* and the Fe (110) surface. *E*_ads_ value of *PTH* followed the trend: *PTH* < Cl^−^ < H_3_O^+^ < H_2_O.

**Table 11:** The output energies calculated by the Monte Carlo simulation for *PTH* in both gas and liquid phases.

Inh.	Phase	E_T_ (kJ/mol)	E_ads_ (kJ/mol)	E_rig.ads_ (kJ/mol)	E_def_. (kJ/mol)	dE_ad_/dN PTH	dE_ad_/dN H_2_O	dE_ad_/dN H_3_O^+^	dE_ad_/dN Cl^−^
***PTH***	**Gas Phase**	−83.974	−247.68	−220.27	−27.41	−247.68	-----	-----	-----
	**Solvent Phase**	−7829.57	−12735.4	−8135.31	−4600.09	−259.614	−33.604	−137.604	−168.601

This pattern suggests that *PTH* molecules can effectively displace the corrosive species, creating an adhering adsorption film with the *CS* surface, which enhances *PTH* mitigation efficiency^32,33^. Also, the *E*_ads_ value of the compound is studied in Table 11. In the aqueous phase, *E*_ads_ is significantly lower than that in the vacuum phase, which indicates the high adsorption capacity of *PTH* molecules on the *CS* surface via the replacement process of water molecules from the surface, forming a protective adsorbed film, consequently suppressing the contact between *the CS* surface and the corrosive surrounding, decreasing the corrosion rate. Additionally, *E*_ads_ value with a -ve sign bolstered the spontaneous adsorption of the investigated *PTH*^112^. Finally, all these observations highlighted the vital role of the investigated *PTH* in *CS* protection via the construction of a defensive film against the destructive particles, consequently decreasing the *CS* corrosion rate. The interpretations of *DFT* and the *MCs* output indices are side by side and agree with the laboratory (gravimetric, electrochemical, and surface examinations) data. These annotations matched with the previously published paper^39^. Also, the inhibition efficiency of the investigated *PTH* inhibitor was compared with previously reported inhibitors, as seen in Table 12, showing its superior mitigation potency at low inhibitor dosage, which is ideal for economical applications.

**Table 12:** Comparison between the Inhibition efficiency of the prepared *PTH* with other similar inhibitors.

Inh.	Conc.	EIS	PDP	Reference
		$\:\eta\:\%$	$\:\eta\:\%$	
*1*	2 × 10^–3^	85.4	81.9	^113^
*Imine-NO*_2_	1 × 10^–3^	89.1	90.0	^114^
*EEMD*	1 × 10^–3^	87.0	88.1	
*INB*	1 × 10^–3^	91.1	90.2	^115^
*IMB*	1 × 10^–3^	91.9	91.5	
*Tetra-Pz-Ortho*	1 × 10^–3^	96.9	97.2	^116^
*Tetra-Pz-Para*		96.2	96.0	
*L3*	1 × 10^–3^	76.83	75.5	^117^
*4-PCPTC*	1 × 10^–3^	89	89	^118^
*SB-CH*_3_	1 × 10^–3^	89.44	89.19	^69^
*SB–OCH*_3_		90.30	89.31	
*S1H*	5 × 10^–3^	----	91	^119^
*Isonitrosoacetanilide*	5 × 10^–3^	79.3	75.7	^120^
*S4*	1 × 10^–2^	92.6	92.2	^121^
*PTH -Sb*	5 × 10^–4^	95.38	95.78	Present study

### Surface examinations (SEM, EDX, AFM, and XPS)

**Fig. 12:**
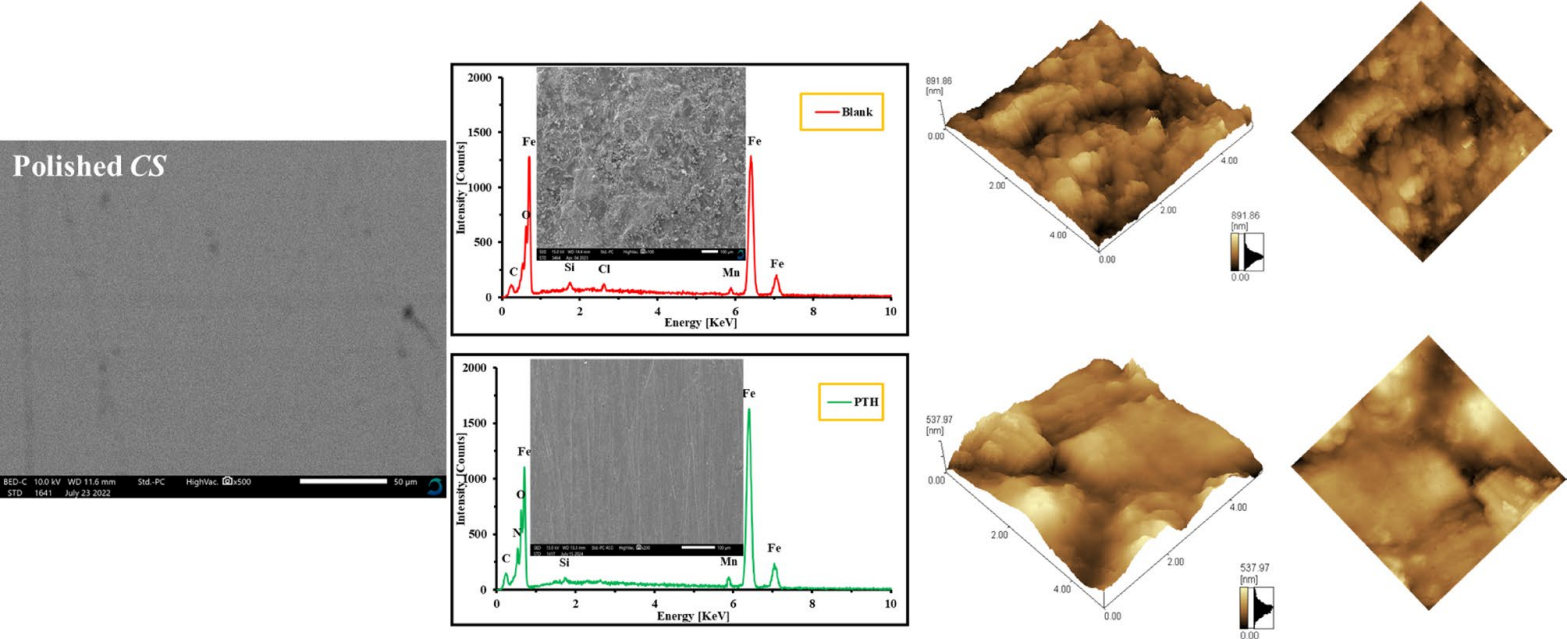
*SEM*, *EDX*, and AFM for *CS* in 1 M HCl, free and containing *PTH* after 10 h immersion time obtained by QUANTA FEG 250-SEM and Park System XE-100 AFM.

The surface of *CS* was examined through SEM, a highly efficient method that complements *EMs*, to assess the corrosive effects of 1.0 M HCl and the protective influence of *PTH* as depicted in Fig. 12. The *CS* morphology after polishing was smooth, while after immersion in 1.0 M HCl without the *PTH* inhibitor, a damage and presence of corrosion products resulting from the aggressive action of HCl^113,114^. The corrosion-inhibiting capability of *PTH* was proved as in Fig. 12, with an improved surface that appeared significantly smoother and largely free from corrosion residues (iron oxides and chlorides) compared to the untreated counterpart^9,115^. This improvement confirmed the formation of a protective adsorption film by *PTH* molecules acting as a shield layer that minimized the direct interaction between the *CS* surface and the corrosive environment^116,117^.

The chemical composition and the distribution of *PTH* film were investigated by EDX studies for determination the elemental distribution (O, Cl, C, Fe, and N) in the absence and presence of *PTH* as depicted in Fig. 12. The untreated sample exhibited chloride (Cl⁻) weight% of 6.38% while, after *PTH* addition, the chloride dropped significantly to 1.23%. The presence of Cl⁻ comes from the adsorbed species or corrosion products such as FeCl_ads_ or FeCl_2_, which previously formed on the *CS* surface, on the uncovered surface, or under the film. While the carbon (C) and oxygen (O) contents were raised from 3.24%, 10.09% and 6.28% to 4.93% respectively. This observation proved the adsorption process of the *PTH* molecules over the *CS* surface, forming a defensive film against the aggressive attack of HCl.

Also, the adsorbed Cl^−^ ions may also behave as a connecting bridge between the metal surface and the protonated organic molecules (*PTH*) to facilitate the adsorption process. The existence of N-peak (1.19%) proved the ability of *PTH* molecules to adhere to *CS* surface, forming a protective barrier layer^118,119^. The notable enhancement of the *Fe* signal in the *EDX* spectra following *PTH* treatment further suggested a surface largely devoid of corrosion byproducts^120^. Also, the surface of *CS* was examined using *AFM* imaging to analyze the alterations in *CS* morphology prior to and following the injection of *PTH* as seen in Fig. 12. The *AFM* images displayed *CS* surface in the untreated solution with a significant damage and very rough surface characterized by large depths and pits formed due to the aggressive action of the HCl solution with average roughness (*R*_*a*_) value of 36.2 nm. Conversely, the *CS* surface after the injection of *PTH* exhibited a more uniform and smoother surface with moderate cracks and pits, with a *R*_*a*_ value of 13.5 nm. The significant reduction in *R*_*a*_ value bolstered that a smoother texture of *CS* is maintained after the injection of the *PTH*. Therefore, it can be concluded that the introduction of *PTH* provided substantial protection to the *CS* in a highly aggressive HCl environment. These findings underscore the efficacy of the tested *PTH* inhibitor in safeguarding the *CS* surface from the detrimental effects of the corrosive agents, facilitated by enhanced *CS* surface coverage with the adsorbed *PTH* molecules and the formation of an insoluble barrier layer that mitigates the corrosion rate of *CS*^63,121–123^.

**Fig. 13:**
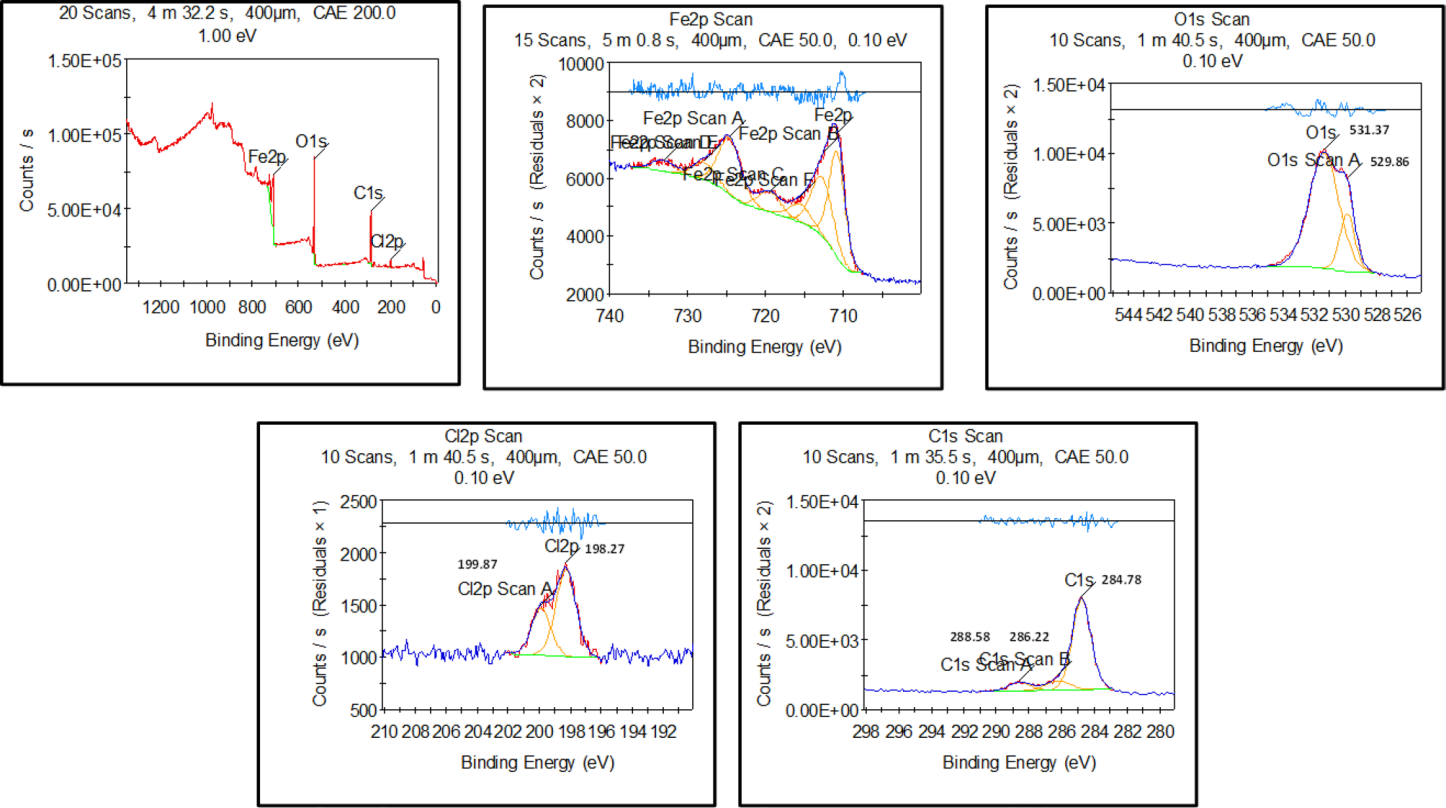
*XPS* analysis of *CS* in blank solution (1 M HCl) after 10 h immersion time.

**Fig. 14:**
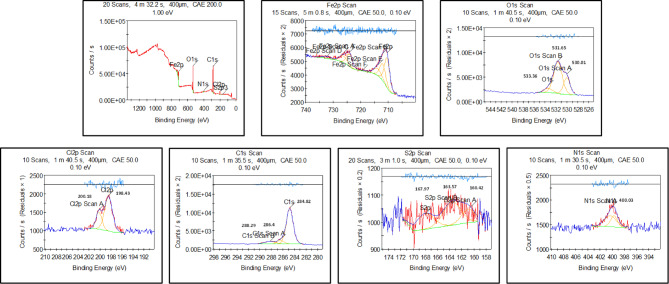
*XPS* analysis of *CS* in 1 M HCl containing *PTH* after 10 h immersion time.

**Fig. 15:**
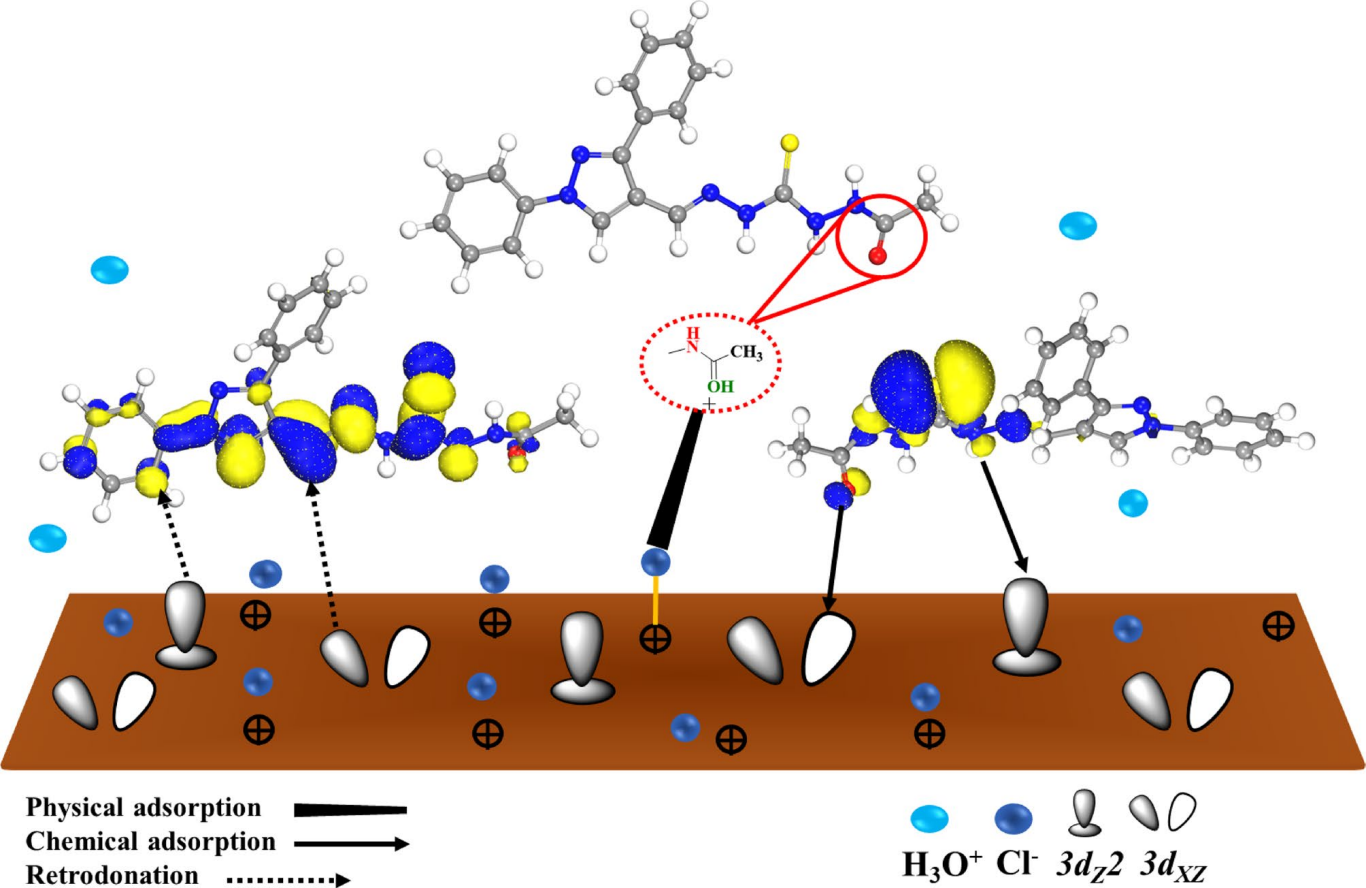
The suggested adsorption mechanism of *PTH* over the *CS* surface obtained by Microsoft PowerPoint.

The *CS* surface was examined in 1.0 M HCl before and after inclusion of *PTH* inhibitor as using the *XPS* tool as depicted in Figs. 13 and 14 for investigating the chemical composition as well as the bonding states of *PTH* upon *CS*, showing C, N, O, S, and Fe elements at the *CS* surface, reflecting *PTH* adsorption. In the absence of *PTH*, the spectral analysis identified Fe, O, and Cl, indicating the corrosion process of *CS*^124^. A detailed examination showed Fe 2p peaks at 710.69 eV and 724.64 eV, alongside binding energies at 712.45, 719.53, 733.18, 727.89, and 715.6 eV, corresponding to the formation of Fe₂O₃, FeOOH, FeCl₃, FeO, and Fe₃O₄, respectively^11,65^. The *CS* surface was modified with *PTH* addition, showing Fe 2p spectrum exhibited a dominant peak at 710.49 eV, signifying substantial inhibitor coverage that effectively minimized direct exposure of Fe to the corrosive HCl medium^125,126^. Additional insights were gained from the O 1 s spectrum, where the unprotected sample displayed peaks at 530.01 eV and 531.65 eV corresponding to Fe₂O₃, Fe₃O₄, and hydrous iron oxides (FeOOH). A secondary peak at 533.36 eV was attributed to adsorbed water or C–O bonds. The noticeable reduction in these oxygen-related signals upon *PTH* introduction suggests a decrease in the formation and dissolution of corrosion products, emphasizing the inhibitor’s shielding effect^127^. The C 1 s spectrum after injection of *PTH* exhibited characteristic peaks at 284.78, 286.22, and 288.58 eV, corresponding to C–C/C = C/C–H bonds, C–O bonds, and a π–π* demonstration satellite feature linked to aromatic systems and oxygen coordination with Fe vacant orbitals. This fact strongly supported the adsorption of *PTH* on the metal surface and the formation of a protective barrier^128^. Finally, the existence of the N 1 s spectrum further confirmed the adsorption of the studied *PTH* onto the *CS* surface supported with N–Fe and N–C peaks at 400.99 eV and 400.92 eV, respectively. All these findings suggested that nitrogen atoms in the *PTH* molecular structure with lone pair electrons actively interact with the vacant d-orbitals of Fe, which enhanced the inhibitor’s adhesion to the metal surface^36,129^. Finally, *XPS* results confirmed that *PTH* adsorbed onto the *CS* surface with a strong chemical bond, which reflected that *PTH* effectively mitigated HCl corrosion^130^. The suggested adsorption mechanism of *PTH* over the *CS* surface was simulated, as illustrated in Fig. 15, which presents the various adsorption modes.

## Conclusion

In this research, a novel *PTH* synthesized from pyrazole-thiocarbohydrazone was assessed for its effectiveness as a potent corrosion inhibitor for *CS* in a harsh 1.0 M HCl environment. The investigated *PTH* inhibitor was simply synthesized *via* an acetylation reaction at room temperature, then chemically confirmed using various spectroscopic analyses such as *IR*, ^*1*^*HNMR*, and elemental analysis. Based on *WL*, the addition of *PTH* demonstrated that *PTH* acted as an effective inhibitor for *CS* corrosion with $\:\eta\:$ increased with its concentration rising till it touched 93.51% and 95.93% after 1 h and 24 h, respectively. In *PDP* measurements, the findings demonstrated that *PTH* performed as a mixed-type inhibitor, retarding both anodic and cathodic reactions with an inhibition efficacy of 95.78% at 5 × 10⁻⁴ M. Also, *EIS* established a significant increase in *CS* polarization resistance till touch 481.7 Ω.cm² at 5 × 10⁻⁴ M, achieving an inhibition efficiency of 95.38% which can be attributed to *PTH* adsorption process onto *CS* surface *via* chemisorption following the Langmuir adsorption isotherm, suggesting the construction of a stable and defensive film with $\:\varDelta\:{G}_{\text{a}\text{d}\text{s}}^{^\circ\:\:}$= −47.2 kJ.mol^− 1^. In addition, the *CS* surface morphology was evaluated using *SEM*,* EDX*, AFM, and *XPS*, confirming the *PTH* effectiveness *via* the construction of a barrier adsorption layer of *PTH* molecules that minimized corrosion rate. *DFT* calculations and *MCs* offered molecular-level insights into the electronic structure and adsorption behavior of *PTH* onto Fe (110), supporting the experimental results. The combined electrochemical, surface, and computational analyses strongly validate the effectiveness of *PTH* as a highly efficient corrosion inhibitor.

## Data Availability

All data generated or analyzed during this study are included in this published article and its supplementary information files.
